# Interactions of *WRKY15* and *WRKY33* transcription factors and their roles in the resistance of oilseed rape to *Sclerotinia* infection

**DOI:** 10.1111/pbi.12838

**Published:** 2017-11-09

**Authors:** Fei Liu, Xiaoxia Li, Meirong Wang, Jing Wen, Bin Yi, Jinxiong Shen, Chaozhi Ma, Tingdong Fu, Jinxing Tu

**Affiliations:** ^1^ National Key Lab of Crop Genetic Improvement, National Center of Rapeseed Improvement in Wuhan College of Plant Science and Technology Huazhong Agricultural University Wuhan Hubei China; ^2^ National Center of Rapeseed Improvement in Wuhan College of Plant Science and Technology Huazhong Agricultural University Wuhan Hubei China; ^3^ Present address: Department of Plant Science University of Manitoba Winnipeg Manitoba Canada

**Keywords:** *Brassica napus*, *BnWRKY15*, *BnWRKY33*, *Sclerotinia sclerotiorum*

## Abstract

WRKY transcription factors are known to participate in the defence responses of higher plants. However, little is known about the roles of such proteins, especially regarding their functions in the resistance of oilseed rape (*Brassica napus*) to *Sclerotinia sclerotiorum*, a necrotrophic fungal pathogen that causes stem rot. In this study, we identified *BnWRKY33* as a *S. sclerotiorum*‐responsive gene that positively regulates resistance to this pathogen by enhancing the expression of genes involved in camalexin synthesis and genes regulated by salicylic acid (SA) and jasmonic acid (JA). We also identified a *S. sclerotiorum*‐responsive region in the promoter of *BnWRKY33*, which we revealed to be a relatively conserved W‐box region in the promoters of homologous genes in different species. Using this *S. sclerotiorum*‐responsive region as bait in a yeast one‐hybrid assay, we identified another WRKY transcription factor, *BnWRKY15*, and observed that both BnWRKY15 and BnWRKY33 could bind to this region. In addition, *BnWRKY15* overexpression simultaneously increased the susceptibility of *B*. *napus* to *S. sclerotiorum* and down‐regulated *BnWRKY33* after different durations of infection. Furthermore, BnWRKY15, which contains a transcriptional repression domain, exhibited reduced transactivation ability and could reduce the transactivation ability of BnWRKY33 in *Arabidopsis* protoplast assays. Therefore, we suggest that the increased susceptibility of *BnWRKY15*‐overexpressing plants results from reduced *BnWRKY33* expression, which is due to the inhibition of BnWRKY33 transcriptional activation by BnWRKY15.

## Introduction

Oilseed rape (*Brassica napus* L.) is an important economic crop that is susceptible to *Sclerotinia sclerotiorum*, which causes sclerotinia stem rot, a notorious disease in oilseed rape that is responsible for significant yield losses. *Sclerotinia sclerotiorum* can infect more than 400 plant species, including many important agronomic crop species (Boland and Hall, 1994). However, little is known about function of the defence‐related genes of oilseed rape against this pathogen.

One area of interest is the role of the WRKY transcription factor (TF) superfamily. The WRKY family is one of the ten largest families of TFs in higher plants (Rushton *et al*., 2010) and comprises 74 members in the model plant *Arabidopsis thaliana* (Ulker and Somssich, 2004). WRKY proteins are classified into three classes on the basis of both the number of WRKY domains and the features of their zinc‐finger‐like motif: group I contains two WRKY domains, and both groups II and III, which have only one WRKY domain, are distinguished by their zinc‐finger‐like motif (Eulgem *et al*., 2000). WRKY proteins play critical roles in both plant development and defence responses (Chen and Chen, 2002; Dang *et al*., 2013; Eulgem and Somssich, 2007; Eulgem *et al*., 2000; Luo *et al*., 2005; Pandey and Somssich, 2009; Qiu *et al*., 2007; Singh *et al*., 2002; Tao *et al*., 2009; Xu *et al*., 2006; Yu *et al*., 2001), and WRKY TFs may also regulate other plant physiological processes (Eulgem and Somssich, 2007; Xie *et al*., 2005). WRKY TFs in *B. napus* were first characterized by Yang *et al*. (2009), and the expression of 16 of 38 cloned *WRKY*s (i.e. *BnWRKY*s) was induced in response to infection by both *S. sclerotiorum* and *Alternaria brassicae*, including the expression of *BnWRKY33* (Yang *et al*., 2009), which enhances the resistance of *B. napus* to *S. sclerotiorum* (Wang *et al*., 2014). Furthermore, because *B. napus* is closely related to *A. thaliana* (Chalhoub *et al*., 2014; Lagercrantz, 1998; Schmidt *et al*., 2001; Wang *et al*., 2011), whose defence responses have been studied thoroughly, the defence responses of *B. napus* can be investigated further using the *Arabidopsis* system as a reference.

All group IId WRKY TFs in *Arabidopsis* (Eulgem *et al*., 2000) negatively regulate plant responses to both biotic and abiotic stresses, with the exceptions of *AtWRKY39* and *AtWRKY21*, whose functions have not yet been reported (Journot‐Catalino *et al*., 2006; Kim *et al*., 2006; Li *et al*., 2010; Vanderauwera *et al*., 2012). Furthermore, elevated expression of the group IId WRKY TF *AtWRKY15* increases the sensitivity of *Arabidopsis* to osmotic and oxidative stresses and impairs mitochondrial stress responses (Vanderauwera *et al*., 2012). Also, the group I WRKY TF *AtWRKY33* (Eulgem *et al*., 2000) improves the resistance of *Arabidopsis* to various necrotrophic fungi, including *Botrytis cinerea* and *Alternaria brassicicola,* but enhances susceptibility to the bacterial pathogen *Pseudomonas syringae* (Zheng *et al*., 2006). In addition, expression of both the jasmonate‐regulated gene *PLANT DEFENSIN 1.2* (*PDF1.2*) and the salicylate‐regulated gene *PATHOGENESIS‐RELATED GENE 1* (*PR‐1*) is altered in *AtWRKY33*‐overexpressing transgenic plants (Zheng *et al*., 2006).

Both AtWRKY25 and AtWRKY33 interact with MAP kinase substrate 1 (MKS1) *in vitro* and are phosphorylated by mitogen‐activated protein kinase 4 (MPK4) (Andreasson *et al*., 2005), a member of the mitogen‐activated protein kinase (MAPK) cascade, which plays pivotal roles in many diverse processes (Meng and Zhang, 2013). In the absence of pathogens, MPK4, AtWRKY33 and MKS1 occur as a complex within the nucleus; however, when activated by *P. syringae* or flagellin, MPK4 phosphorylates MKS1 and releases MKS1 and WRKY33 (Qiu *et al*., 2008). MKS1 is a member of the VQ motif‐containing proteins (VQ proteins), which play important roles in plant development and stress‐related processes by acting as cofactors of WRKY TFs (Cheng *et al*., 2012). AtWRKY33 subsequently activates the expression of *PHYTOALEXIN DEFICIENT 3* (*PAD3*) and *Cytochrome P450 71A13* (*CYP71A13*) (Qiu *et al*., 2008), both of which are involved in camalexin (3‐thiazol‐2′‐yl‐indole) synthesis and resistance to the fungal pathogen *A*. *brassicicola* (Nafisi *et al*., 2007; Schuhegger *et al*., 2006; Zhou *et al*., 1999). Moreover, many direct targets of AtWRKY33 were recently reported to be involved in hormone signalling and phytoalexin biosynthesis (Liu *et al*., 2015). Therefore, *AtWRKY33* might modulate and perceive upstream signals and regulate camalexin synthesis in response to pathogens. Chromatin immunoprecipitation (CHIP) using an anti‐all‐WRKY serum has demonstrated that the *AtWRKY33* promoter is a potential target of other WRKY TFs (Lippok *et al*., 2007), but no investigations have definitely characterized these WRKY TFs that function upstream of *WRKY33*. However, AtWRKY33 itself can bind to its own promoter *in vivo* and functions downstream of mitogen‐activated protein kinase 3/ mitogen‐activated protein kinase 6 (MPK3/MPK6), potentially forming a positive feedback regulatory loop (Mao *et al*., 2011). In the present study, we investigated a novel upstream regulator, *BnWRKY15*, which showed a negative role in response to *S. sclerotiorum*.

Regarding the interaction of *B. napus* and *S. sclerotiorum*, studies have focused on transcriptional and translational changes in response to pathogen infection (Garg *et al*., 2013; Liang *et al*., 2008; Wu *et al*., 2016; Yang *et al*., 2007; Zhao *et al*., 2007), including the roles of *BnWRKY33* in response to *S. sclerotiorum* (Wang *et al*., 2014; Yang *et al*., 2009). However, studies on upstream regulators of *BnWRKY33* in *B. napus* are scarce. Therefore, to provide more information about the important resistance gene *WRKY33*, we investigated the roles of *BnWRKY33* and its upstream regulator in response to *S. sclerotiorum* as well as relationships between them.

## Results

### Expression analysis of *BnWRKY33*

**Figure 1:**
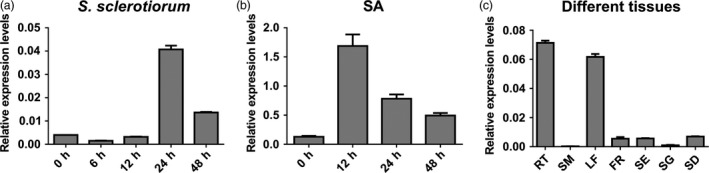
Expression analysis of *BnWRKY33* in leaves with the treatment of *Sclerotinia sclerotiorum* and salicylic acid (SA) as well as in different tissues. (a) Relative *BnWRKY33* expression levels were quantified in *Brassica napus* (Ning RS‐1) using quantitative RT‐PCR at 0, 6, 12, 24 and 48 h after inoculation with *S. sclerotiorum*. Each fifth leaf of one‐month‐old plants was used for infection. (b) *BnWRKY33* expression levels after treatment with SA. Each fifth leaf of three‐week‐old plants treated with SA was sampled at different time points and used for RNA purification. (c) Expression analysis of *BnWRKY33* in different tissues including roots (RT) (roots from one‐week‐old Ning RS‐1 plants), stems (SM) (peduncle‐growing period), leaves (LF) (the third leaf of two‐week‐old plants), flowers (FR) (peduncle‐growing period), siliques (SE) (ten days after flowering), seedlings (SG) (one‐week‐old plants) and seeds (SD) (thirty days after flowering). The values and error bars indicate means ± standard error (*n* = 3).

**Figure 2:**
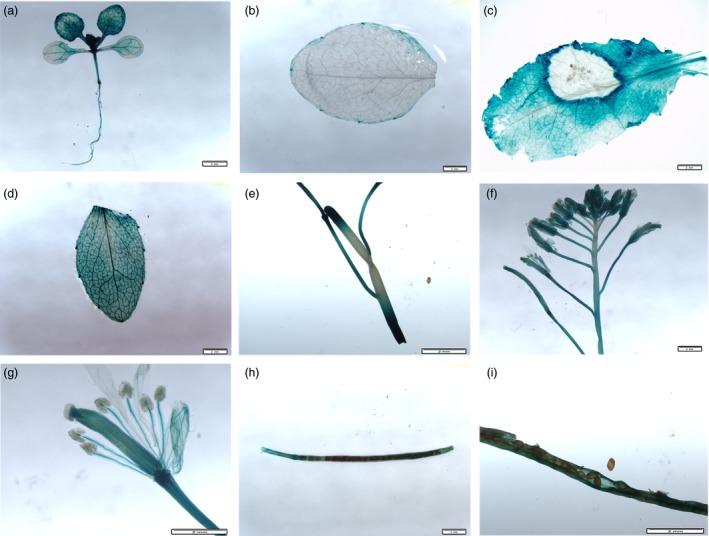
Analysis of the expression of the β‐glucuronidase (GUS) reporter gene driven by the *BnWRKY33* promoter in transgenic *Arabidopsis* plants. GUS histochemical staining of transgenic *Arabidopsis* is shown in different tissues: (a) seedlings (two‐week‐old plants) and (b) rosette leaves (one‐month‐old plants), each without an elicitor; (c) rosette leaves (one‐month‐old plants) inoculated with *S. sclerotiorum* for 24 h; (d) rosette leaves treated with SA for 12 h; (e) detached stems; (f) flowers; (g) buds; and (h and i) siliques. Scale bar, 2 mm.

Quantitative RT‐PCR (qPCR) using cDNA from *S. sclerotiorum*‐inoculated leaves indicated that *BnWRKY33* expression peaked at 24 h after inoculation but decreased at 48 h (Figure 1a); this TF was also induced by salicylic acid (SA) treatment (Figure 1b). Expression levels were also assayed across different tissues: the expression levels were greatest in roots and leaves; lowest in stems and seedlings; and moderate in flowers, siliques and seeds (Figure 1c). Furthermore, using native promoter‐β‐glucuronidase (GUS) fusion constructs in two‐week‐old *A. thaliana* (Col‐0) seedlings, GUS activity was detected in different tissues but was primarily distributed in newly growing or young leaves as well as in the roots (Figure 2a); these observations were largely in accordance with the qPCR results. In addition, elevated GUS activity was observed around the infection sites of *S. sclerotiorum*‐infected leaves as well as in SA‐treated leaves (Figure 2b, c and d). In rosette leaves of plants grown under normal conditions, GUS activity was observed only on the edge of the rosette leaf blades (Figure 2b), and less GUS activity was observed in the stem, except at the two ends wounded during sampling (Figure 2e). In addition, during floral development, GUS activity was observed in the sepals, petals, filaments and styles (Figure 2f and g) and in the walls of siliques (Figure 2h and i).

### Resistance of *BnWRKY33*‐overexpressing plants to *S. sclerotiorum*

**Figure 3:**
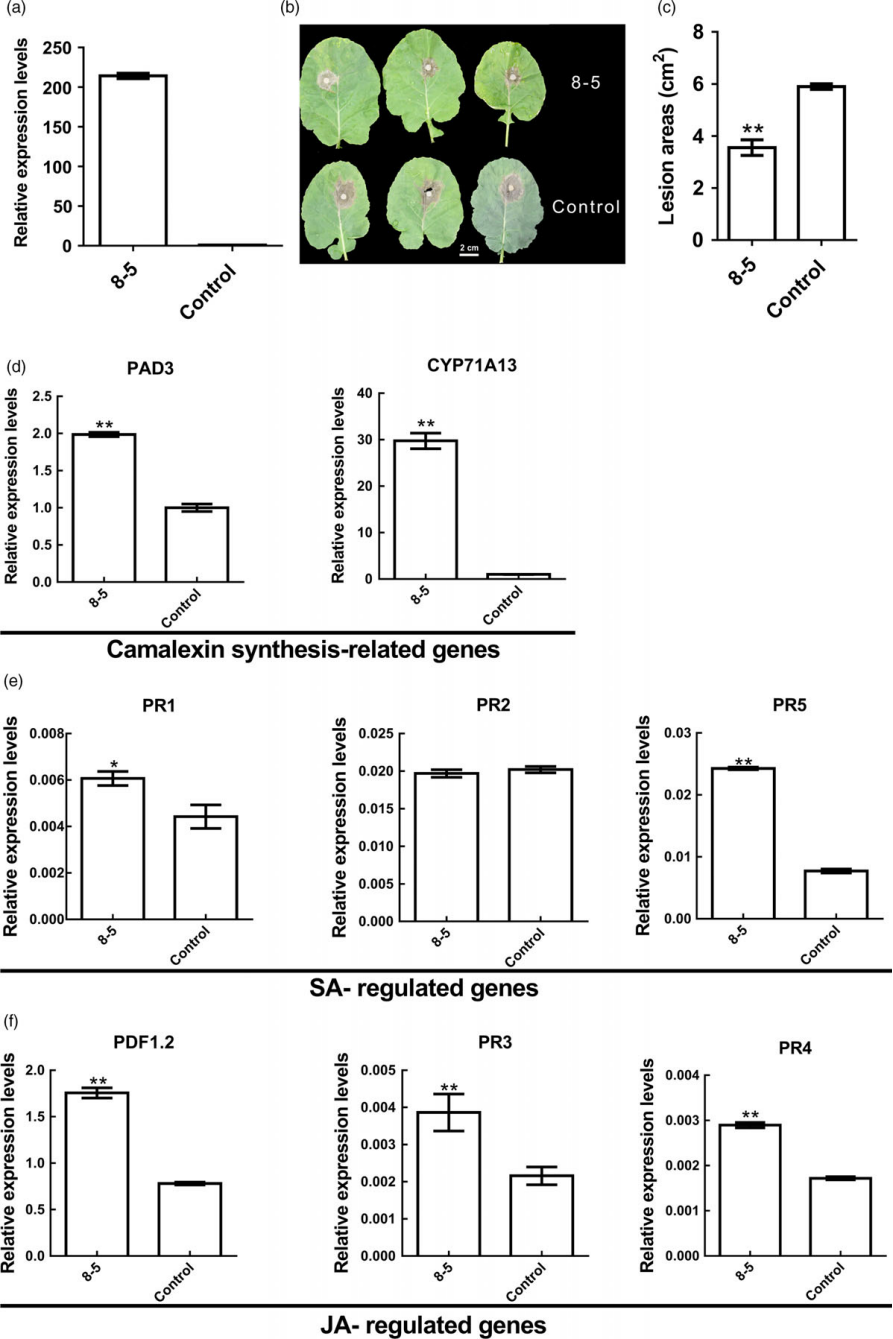
Effects of *BnWRKY33* overexpression on the resistance and expression of defence‐related genes. (a) *BnWRKY33* expression levels were quantified in transgenic plants and controls (Westar). (b) The lesions of detached leaves from T_2_ plants were imaged at 48 h after inoculation. Scale bar, 2 cm. (c) Areas of lesions in the leaves were measured 48 h postinoculation. Three plants of transgenic and control lines each were used for assays. (d) Expression levels of camalexin synthesis‐related genes in *BnWRKY33*‐overexpressing lines (8‐5) and the control (Westar). (e) Expression difference of SA‐regulated genes between *BnWRKY33*‐overexpressing lines and the control. (f) Relative expression levels of JA‐regulated genes in *BnWRKY33*‐overexpressing lines and the control. The relative expression values were from three biological replicates. Single asterisks (*) indicate significance at *P *<* *0.05 (Student's *t*‐test). Double asterisks (**) indicate highly significant differences (*P *<* *0.01; Student's *t*‐test). The error bars indicate standard error.

To study the role of *BnWRKY33* in the resistance to *S. sclerotiorum*, we cloned *BnWRKY33* from cDNAs of *B. napus* (Ning RS‐1) and mapped the gene onto linkage group A05 using the ‘Tapidor‐NY7’ (TN) doubled‐haploid (DH) population (Qiu *et al*., 2006) in conjunction with intron polymorphism (IP) molecular marker 33‐56yh (Figure S1). Furthermore, *BnWRKY33* showed more similarity with the A05 copy than with paralogs on other chromosomes when *BnWRKY33* sequences were used as BLAST queries against the *B. napus* genome (http://www.genoscope.cns.fr/brassicanapus/). We then overexpressed this copy in the *B. napus* cultivar Westar; most of the transformed parental (i.e. T_0_ generation) lines exhibited enhanced resistance (Table S1). Of all the T_0_ lines, line 33‐32 had the greatest levels of both expression and resistance (Figure S2 and Table S1), and the T_2_ generation line 8‐5, which originated from line 33‐32, consistently showed significantly greater resistance compared with that of the Westar control (Student's *t*‐test, *P *<* *0.01; Figure 3b and c). The expression level of *BnWRKY33* in line 8‐5 was also consistently elevated (Figure 3a). The *BnWRKY33*‐overexpressing plants also exhibited elevated expression of *PAD3* and *CYP71A13* (Figure 3d); the SA‐regulated genes *PR1* and *PR5* (Figure 3e); and the three jasmonic acid (JA)‐regulated genes, *PDF1.2*,* PR3* and *PR4* (Figure 3f).

### Identifying the *S. sclerotiorum*‐responsive region and its interactive proteins

**Figure 4:**
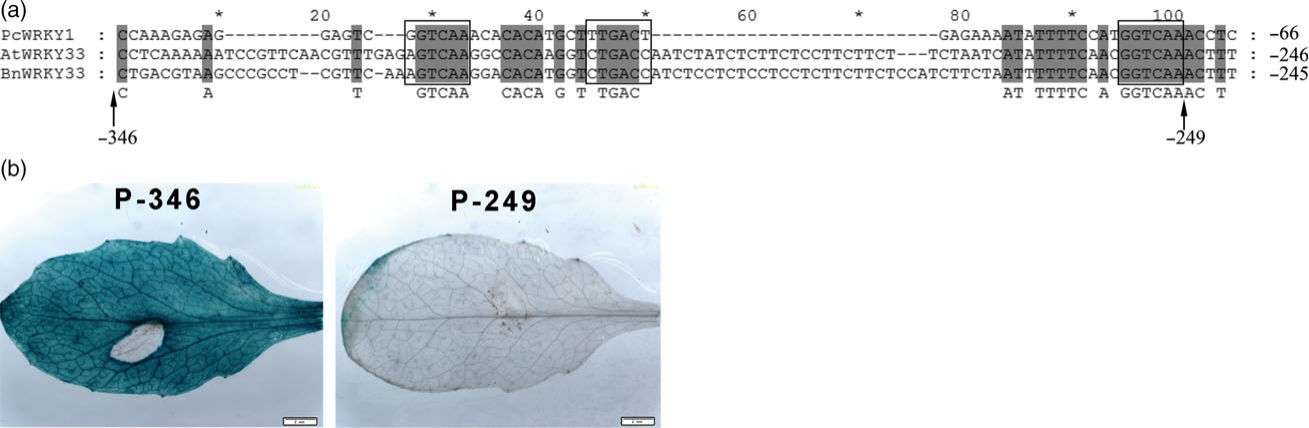
Identification of the *Sclerotinia sclerotiorum*‐responsive region in the promoter of *BnWRKY33*. (a) Sequence alignment of the promoter regions of *BnWRKY33*,* AtWRKY33* and *PcWRKY1*. The numbers −346 and −246 indicate the truncated location in the promoter. The black box represents the W‐box region in the promoter. (b) *Sclerotinia sclerotiorum*‐induced β‐glucuronidase (GUS) expression in 1‐month‐old rosette leaves of transgenic *Arabidopsis* carrying the truncated promoters P‐346‐GUS and P‐249‐GUS at 24 h after inoculation with *S. sclerotiorum*. Scale bar, 2 mm. Similar results were obtained for three independent replicate experiments that involved the use of different transgenic lines.

To isolate the *S. sclerotiorum*‐responsive region of the *BnWRKY33* promoter, we predicted the *cis*‐elements of the promoter sequence and identified three W‐box elements in the region from −249 to −346 (Figure S3), designated as the 33box. In addition, this region shared relatively conserved W‐boxes among the promoters of the *BnWRKY33* homologs *AtWRKY33* and *PcWRKY1* (Lippok *et al*., 2007; Turck *et al*., 2004) (Figure 4a). We then fused the GUS gene to the truncated promoter either with (P‐346) or without (P‐249) the region containing the three W‐box elements, generating the constructs P‐346‐GUS and P‐249‐GUS, respectively (Figure 4a). Only the transgenic plants carrying the P‐346‐GUS construct exhibited strong GUS activity upon infection with *S. sclerotiorum*, whereas those transformed with P‐249‐GUS did not (Figure 4b). In addition, plants harbouring P‐249‐GUS showed very weak staining on the margins of rosette leaves without *S. sclerotiorum*, and plants containing P‐346‐GUS also displayed similar GUS staining on the margins of rosette leaves (Figure S4b and c). GUS staining in other tissues of *Arabidopsis* containing P‐249‐GUS displayed similar patterns as those of the native promoter (Figure S4). Thus, we concluded that the 33box region containing the W‐box elements was responsible for the transcriptional activation of *BnWRKY33* by *S. sclerotiorum* infection.

To identify the proteins that interact with the pathogen‐responsive region (33box) and that function upstream of *BnWRKY33*, we performed yeast one‐hybrid assays using the 33box region as bait to screen approximately 1.035 × 10^6^ independent transformants from a cDNA library of *S. sclerotiorum*‐infected *B. napus* leaves. Of the fifty‐two clones (Table S3) that exhibited homology to *Arabidopsis* sequences, one (*BnWRKY15*) was identified as the homolog of *AtWRKY15* according to the BLASTN analysis (Figure S5). As this region contains three W‐box elements, the W‐box‐binding protein BnWRKY15 was preferentially considered for subsequent research.

**Figure 5:**
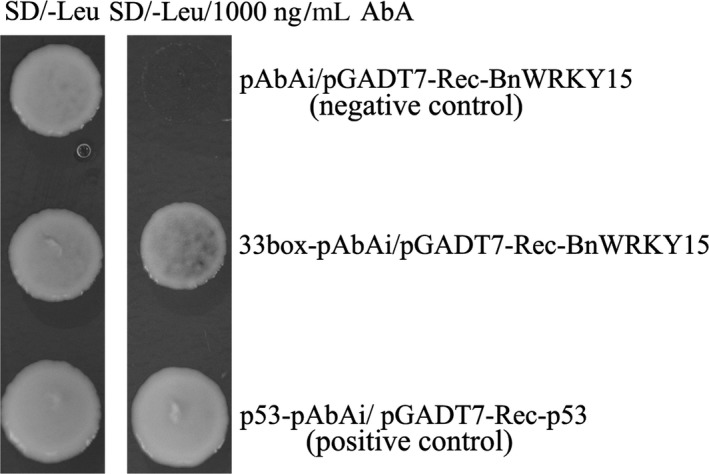
Yeast one‐hybrid assay for detecting specific interactive effects between 33box and BnWRKY15. The bait strain generated by integrating linearized 33box‐pAbAi into the genome of the Y1HGold yeast strain was transformed with the effector plasmid PGADT7‐Rec‐BnWRKY15 to validate the interaction. Y1HGold integrated with linearized empty pAbAi was transformed with the BnWRKY15 effector plasmid pGADT7‐Rec‐BnWRKY15 and served as a negative control. In addition, a positive control was generated by cotransforming the pGADT7‐Rec vector and p53 control into the Y1HGold, chromosome of which was integrated with the linearized p53‐pAbAi control plasmid. The normal growth of three Y1HGold yeast strains on SD/‐Leu plates in the absence of AbA indicated that the yeast growth status was healthy and unaffected by other factors (left), whereas only the bait strain containing the effector plasmid pGADT7‐Rec‐BnWRKY15 and the positive control showed growth on SD/‐Leu containing 1000 ng/mL aureobasidin A, suggesting specific interaction between BnWRKY15 and 33box (AbA; right).

After the reporter strain (containing 33box‐pAbAi) and null reporter strain (containing an empty pAbAi) were retransformed with the effector plasmid pGADT7‐Rec‐BnWRKY15, both sets of cells plus a positive control (p53) grew on synthetic defined (SD)/‐Leu medium in the absence of aureobasidin A (AbA; Figure 5, left), and the growth of both the 33box‐pAbAi reporter yeast cells containing the BnWRKY15 effector and the positive control (p53) was not inhibited on SD/‐Leu medium containing 1000 ng/mL AbA (Figure 5, right). However, the pAbAi reporter cells containing the BnWRKY15 effector failed to grow (Figure 5, right), suggesting that BnWRKY15 can bind to the *BnWRKY33* W‐box region.

### Interaction of 33box with BnWRKY15 and BnWRKY33

**Figure 6:**
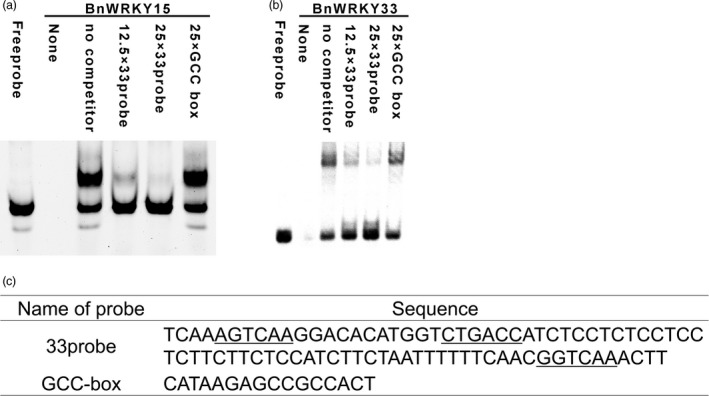
Electrophoretic mobility shift assay showing the binding of BnWRKY15 or BnWRKY33 proteins to the *Sclerotinia sclerotiorum*‐responsive region. (a) BnWRKY15 6× His fusion proteins bind specifically to the *S. sclerotiorum*‐responsive promoter region (designated as the 33probe) *in vitro*. (b) *In vitro* binding of BnWRKY33 6× His fusion proteins to the 33probe was validated using EMSA. The labelled probes plus 0‐, 12.5‐ or 25‐fold excess unlabelled 33probe and unlabelled GCC‐box probes were used for binding assays. Labelled probes or recombinant His tag proteins served as controls. (c) Sequences of the 33probe (W‐boxes are underlined) and GCC‐box.

We subsequently validated the binding of the region by BnWRKY15 by performing an electrophoretic mobility shift assay (EMSA) using recombinant proteins and probes, and the mixture excluding recombinant proteins or probes were loaded and served as controls. BnWRKY15 6 × His fusion proteins could bind to the DNA probes (Figure 6a and c), and the DNA‐binding intensity of BnWRKY15 fusion proteins decreased due to competition from unlabelled probes but not from the GCC‐box (Figure 6a and c), which suggests BnWRKY15 exhibits binding specificity.

**Figure 7:**
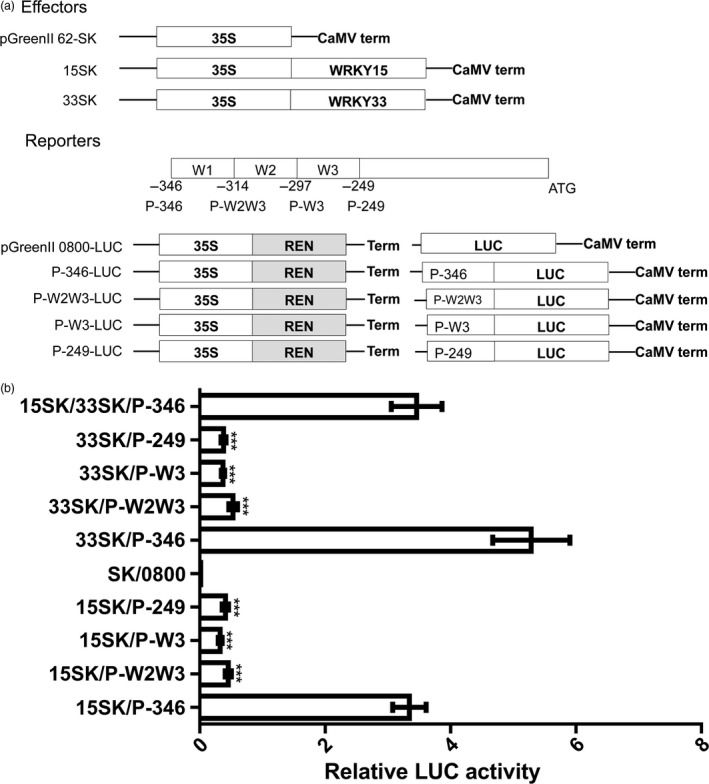
Defined region bound for activation by BnWRKY15 and BnWRKY33 obtained by *Arabidopsis* protoplast transient assays. (a) Schematic representation of effector and promoter reporter constructs used in *Arabidopsis* protoplast transient assays. The open reading frame of *BnWRKY15* or *BnWRKY33* was inserted into pGreenII 62‐SK to generate 15SK or 33SK plasmids as effectors. The promoter regions containing the first W‐box, second W‐box and third W‐box were designated regions W1, W2 and W3, respectively. Different promoter regions (P‐346, P‐W1W2, P‐W3 and P‐249) of *BnWRKY33* fused with the firefly *luciferase* (LUC) gene were used as reporters. The location of each truncated promoter is indicated above the name of each promoter. Empty pGreenII 62‐SK plasmids and empty pGreenII 0800‐LUC construct were cotransformed into protoplasts, serving as a negative control. REN refers to the *Renilla luciferase* gene, which served as an internal control. LUC refers to the *firefly luciferase* gene. (b) The abilities of BnWRKY15 and BnWRKY33 to bind different promoter regions of *BnWRKY33* are indicated by the relative LUC activities, which were calculated by comparing LUC activities to REN activities. The effectors and a reporter were cotransfected. P‐346, P‐W2W3, P‐W3 and P‐249 represent different reporter plasmids that contain different inserted promoter regions, as indicated in (a). SK and 0800 refer to empty effector and empty reporter plasmids. The mark on the y‐axis indicates different combinations of effector and reporter plasmids, and x‐axis represents LUC enzyme activities of these combinations in *Arabidopsis* protoplasts. The data represent the means ± standard errors (*n* ≥ 3). Statistical analyses were performed using Student's *t*‐test: ***P <* *0.001.

In addition, because AtWRKY33 can bind to its own promoter (Mao *et al*., 2011), we used EMSA to investigate whether this phenomenon occurs in *B. napus*. We observed that BnWRKY33 6× His fusion proteins could specifically bind to the pathogen‐responsive promoter region (Figure 6b and c). We also verified our results *in vivo* using an *Arabidopsis* protoplast transient assay in which the P‐346 region (−346 to −1 bp), which included all three W‐box elements (Figure 7a); the P‐W2W3 region (−314 to −1 bp), in which the first W‐box was deleted (Figure 7a); the P‐W3 region (−297 to −1 bp), in which the first and second W‐box elements were deleted (Figure 7a); and the P‐249 region (−249 to −1 bp), in which all three W‐box elements were deleted (Figure 7a), were fused with the *LUC* gene to generate constructs to determine the W‐box region responsible for binding by BnWRKY15 and BnWRKY33 (Figure 7a). *BnWRKY15* overexpression transcriptionally activated the LUC reporter only under the control of the P‐346 promoter (Figure 7b), whereas the activity was abolished when the P‐W2W3, P‐W3 or P‐249 promoter sequence was used. Thus, our results suggest that the first W‐box is necessary for activating the expression of the reporter gene by BnWRKY15 (Figure 7b).

Similar results were obtained in experiments using *BnWRKY33*, which suggests that the first W‐box of its promoter is indispensable for the activation of the reporter gene by BnWRKY33 (Figure 7b). However, when *BnWRKY15* and *BnWRKY33* were co‐expressed with P‐346 constructs under the control of the cauliflower mosaic virus (CaMV) 35S promoter, the overexpression of the two *BnWRKY*s together in *Arabidopsis* protoplast cells resulted in LUC activity levels that were similar to those induced by the overexpression of *BnWRKY15* alone (Figure 7b).

**Figure 8:**
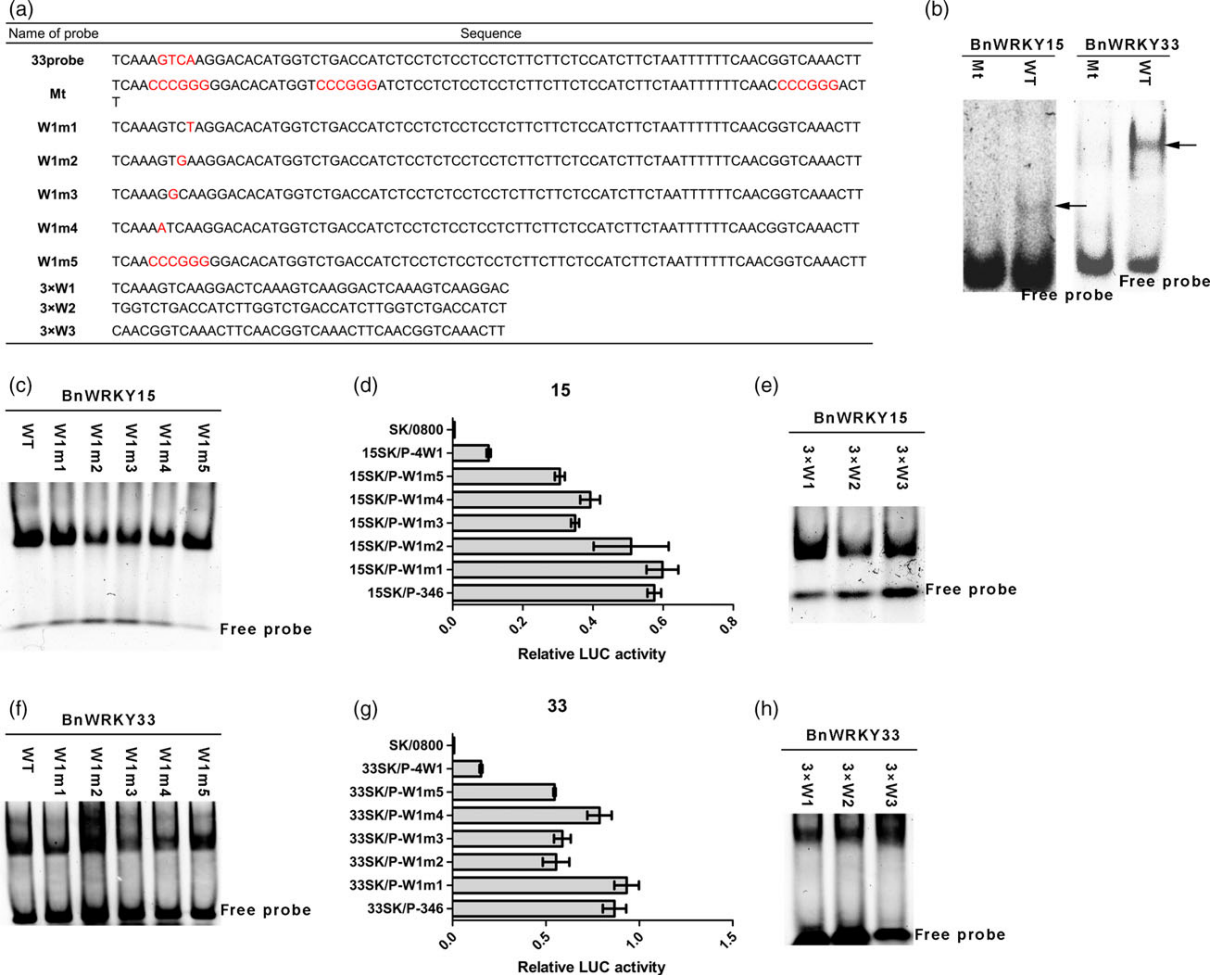
Binding activity of BnWRKY15 and BnWRKY33 for the three W‐box elements in *Brassica napus*. (a) Probes with either the first W‐box mutated or all three W‐box elements mutated were used in electrophoretic mobility shift assays. The W‐box element, TGAC core and mutated base are indicated in red. (b) The binding of the 33probe (WT) and Mt probe (with three W‐boxes mutated, as indicated in (a)) by BnWRKY15 and BnWRKY33 6× His fusion proteins. (c and f) The binding of the 33probe (WT) and mutated probes (sequences indicated in (a)) with the BnWRKY15 or BnWRKY33 6× His fusion proteins. (d and g) Relative luciferase activity from the transient expression analysis of P‐346 and different W1‐mutated promoter reporter plasmids cotransformed with 15SK or 33SK plasmids. The mutations of different W1‐mutated promoters correspond to the sequences of mutated probes in (a). A promoter containing four repeats of the first W‐box in place of the three W‐box regions is designated P‐4W1. The y‐axis represents different combinations of different effector plasmids and reporter plasmids. SK/0800 indicates a negative control (null effector plasmids and null reporter plasmids). The data represent the means ± standard errors (*n* ≥ 3). (e and h) The binding of three repeats of the first, second or third W‐box with BnWRKY15 or BnWRKY33 fusion proteins. Sequences of probes are as indicated in (a).

To confirm whether binding to this region by BnWRKY15 and BnWRKY33 is mediated by W‐boxes, we mutated all three W‐box elements (Mt probe) for EMSA (Figure 8a). The mutation of these sites in the probes abolished their binding by the two BnWRKYs (Figure 8b), which suggests that the three W‐boxes are responsible for binding to this region by the two BnWRKYs. We also used EMSA to investigate whether the first W‐box element was the only site bound by BnWRKY15 and BnWRKY33. To generate mutated probes, we mutated the four base pairs of the core site (TGAC) in the first W‐box, as shown in Figure 8a, and all five forms of W‐box‐mutated probes were bound by either BnWRKY15 or BnWRKY33 (Figure 8c and f). Furthermore, when the corresponding five forms of W‐box‐mutated promoters (P‐W1m1, P‐W1m2, P‐W1m3, P‐W1m4 and P‐W1m5) were used in the *Arabidopsis* protoplast transient assays, activation of the LUC reporter by the two BnWRKYs was similar. Also the *LUC* expression values for the P‐W1 m1‐LUC construct were nearly identical to those of P‐346 (Figure 8d and g), whereas the expression values obtained for the P‐W1m2‐LUC, P‐W1m3‐LUC, P‐W1m4‐LUC and P‐W1m5‐LUC constructs were lower (Figure 8d and g). However, the lowest level of reporter gene expression was observed for the P‐4W1‐LUC construct, in which the responsive region containing the W1, W2 and W3 elements was replaced with four repeats of the W1 element (Figure 8d and g). Thus, even though the W1 element could still be the only W‐box responsible for transcriptional activation, our results suggest that the first W‐box is not the only W‐box bound by the two BnWRKYs. To ascertain whether the other two W‐box elements could be bound by the two BnWRKYs, we examined the binding of the two BnWRKYs to the three individual W‐box elements (Figure 8e and h) and observed that both BnWRKYs could bind to all three W‐box elements.

### Transcriptional activation of *BnWRKY15* and *BnWRKY33*

**Figure 9:**
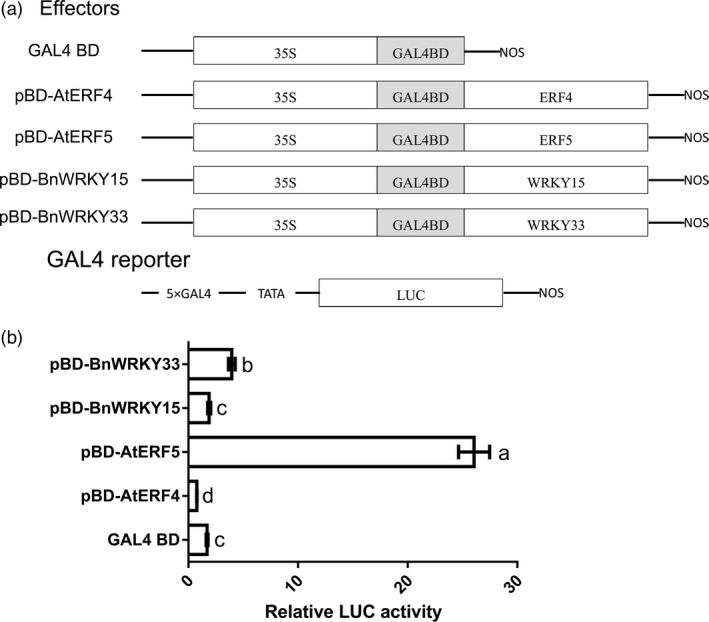
Transcriptional activation of BnWRKY15 and BnWRKY33 in *Arabidopsis* protoplasts. (a) Schematic representation of effector and reporter constructs used in *Arabidopsis* protoplast transient assays. The open reading frames of *BnWRKY15* and *BnWRKY33* were fused to the GAL4 DNA‐binding domain and used as effectors. The transcriptional activation abilities of *AtERF4* and *AtERF5*, which were manifested as a transcriptional activator and a transcriptional repressor, respectively, served as controls. The GAL4 BD effector served as a negative control. (b) Relative luciferase activities were measured after cotransfection of protoplasts with different combinations of reporter and effector plasmids. The results represent the means ± standard errors (*n* = 3). Significantly different values (*P *<* *0.05) according to Tukey's test (ANOVA) are marked with different letters.

The transcriptional activation abilities of *BnWRKY15* and *BnWRKY33* were analysed using a dual‐luciferase reporter assay system, in which *Arabidopsis* protoplasts were cotransformed with pBD‐BnWRKY effector plasmids that contained *BnWRKY* coding regions fused with the GAL4 DNA‐binding domain (Figure 9a) and the firefly *LUC* gene was fused with GAL4 binding sites and a mini‐35S promoter of CaMV, as GAL4 reporter (Figure 9a). The positive control (pBD‐AtERF5) strongly activated the reporter gene, whereas the negative control (pBD‐AtERF4) markedly repressed it (Figure 9b). Furthermore, the reporter was moderately activated by pBD‐BnWRKY33, and the activation by pBD‐BnWRKY15 was similar to that of the GAL4 BD control but greater than that of the pBD‐AtERF4 negative control (Figure 9b). This suggests that BnWRKY33 is a transcriptional activator and that BnWRKY15 is a weak transcriptional repressor.

To identify the activation and repression domains of BnWRKY33 and BnWRKY15, we generated a series of deletion constructs (pBD‐33/376, pBD‐33/276, pBD‐33/176 and pBD‐33/76 for BnWRKY33; pBD‐15/219, pBD‐15/119 and pBD‐15/19 for BnWRKY15; Figure S6a and b). Co‐expression of the effector pBD‐33/376 and reporter plasmids (Figure S6c) resulted in an approximately 50% increase in reporter gene expression compared with that observed with the pBD‐BnWRKY33 construct (Figure S6d). When the region was truncated to 276 or 176 bp (Figure S6a), the relevant effector (pBD‐33/276 and pBD‐33/176) reduced the expression of the reporter gene by approximately 50% compared with that of the effector pBD‐33/376 (Figure S6d). At the same time, the effector pBD‐33/76 increased the reporter gene expression by approximately 50% compared with that observed with the effector pBD‐33/176 (Figure S6d). The truncated effectors pBD‐15/219 and pBD‐15/119 reduced the reporter gene expression by approximately 50% compared with that of the pBD‐BnWRKY15 effector (Figure S6d), whereas the effector pBD‐15/19 increased the expression of the reporter gene to a level similar to that of the control GAL4 BD (Figure S6). In conclusion, the transcriptional activation and repression domains existed in both BnWRKY15 and BnWRKY33.

### Subcellular localization of BnWRKY15 and BnWRKY33

To verify the localization of the two TFs, we fused the coding regions of *WRKY33* and *WRKY15* to the green fluorescent protein (GFP) gene and cotransformed marker constructs into *Arabidopsis* protoplasts using polyethylene glycol (PEG)/calcium‐mediated transformation (Yoo *et al*., 2007). The two recombinant proteins were located exclusively in the nuclei of *Arabidopsis* protoplasts, where they were colocalized with the marker protein (Figure S7). These results indicate that both BnWRKY15 and BnWRKY33 were localized in the nucleus, supporting the roles of TFs as transcriptional regulators.

### Expression analysis of *BnWRKY15*

**Figure 10:**
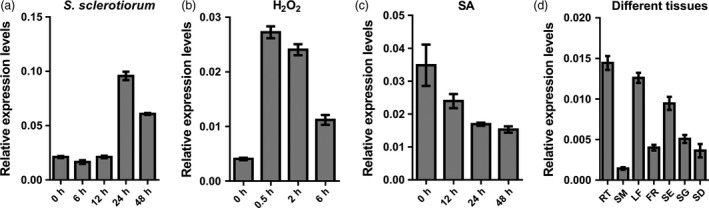
Expression of *BnWRKY15* in leaves treated with different elicitors or in different tissues. (a) Expression levels of *BnWRKY15* in leaves postinfected with *Sclerotinia sclerotiorum* for different durations. (b) Relative expression levels of *BnWRKY15* in leaves treated with H_2_O_2_. (c) *BnWRKY15* expression was determined in leaves treated with salicylic acid. (d) Expression levels of *BnWRKY15* in different tissues, including roots (RT), stems (SM), leaves (LF), flowers (FR), siliques (SE), seedlings (SG) and seeds (SD). The values and error bars indicate means ± standard errors (*n* = 3).

Because *BnWRKY15* was identified and selected from a *S. sclerotiorum*‐infected leaf cDNA library, we investigated via qPCR whether the expression of *BnWRKY15* was affected by infection. *BnWRKY15* transcript abundance peaked at 24 h after inoculation (Figure 10a), and its expression increased at 0.5 h after treatment with H_2_O_2_ (Figure 10b). In addition, the expression levels of *BnWRKY15* gradually decreased after SA treatment (Figure 10c). Expression analysis across different tissues showed that *BnWRKY15* was mainly expressed in the roots and young leaves as well as in siliques (Figure 10d). Accordingly, in response to treatment with *S. sclerotiorum* and H_2_O_2_, strong GUS staining was detected in the rosette leaves of transgenic plants that contained the *BnWRKY15* promoter‐GUS fusion construct (Figure S8c and e), but weak staining was observed at 48 h after SA treatment (Figure S8d). However, GUS staining in transgenic plants was stronger in roots, young leaves and pod epidermis (Figure S8a, i and j), which was similar to that of the *BnWRKY33* promoter, as described above (Figure 2).

### Susceptibility of *BnWRKY15*‐overexpressing plants to *S. sclerotiorum*

**Figure 11:**
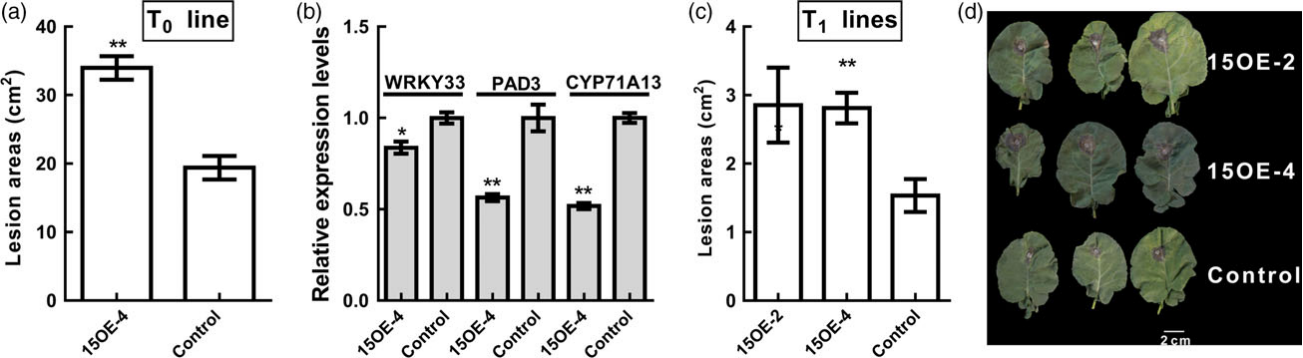
Influence of *BnWRKY15* overexpression on the susceptibility of *Brassica napus* to *Sclerotinia sclerotiorum*. (a) *Sclerotinia sclerotiorum* resistance assays were performed using leaves detached from *BnWRKY15*‐overexpressing plants (T_0_ line, 15OE‐4) and control (Westar) plants. (b) The expression levels of *BnWRKY33*,*PAD3* and *CYP71A13* between the T_0_ line 15OE‐4 and the control (Westar) were compared using quantitative RT‐PCR. (c) To validate the heritability of resistance, two different T_1_ lines (15OE‐2 and 15OE‐4) were used for resistance assays using detached leaves, and lesion areas were measured and imaged at 48 h after inoculation (*n* = 3). Asterisks (* and **) denote significant (*P *<* *0.05) and highly significant (*P *<* *0.01) differences between the transgenic line and the control (Westar), and the error bars indicate standard error (*n* = 3).

By transforming *B. napus* (Westar) with a construct that contained the *BnWRKY15* coding region driven by the CaMV 35S promoter, we obtained eleven T_0_ plants. *BnWRKY15* expression was quantified using qPCR (Figure S9), and T_0_ line 15OE‐4 was selected for resistance assays (line 15OE‐16, which presented the greatest expression level, did not grow well and produced no seeds). After the plants were infected with *S. sclerotiorum*, T_0_ line 15OE‐4 had larger lesion areas than did control plants after infection (Figure 11a), which indicates that *BnWRKY15* overexpression increased the susceptibility of plants to infection. In addition, the expression of *BnWRKY33*,* PAD3* and *CYP71A13* was repressed in *BnWRKY15*‐overexpressing plants (Figure 11b). To confirm the susceptibility of *BnWRKY15*‐overexpressing lines, the T_1_ generation of lines 15OE‐2 and 15OE‐4 were also used to assay the resistance to *S. sclerotiorum*; the results were consistent with those of the T_0_ generation (Figure 11c and d).

### *BnWRKY15‐*mediated modulation of *BnWRKY33*

**Figure 12:**
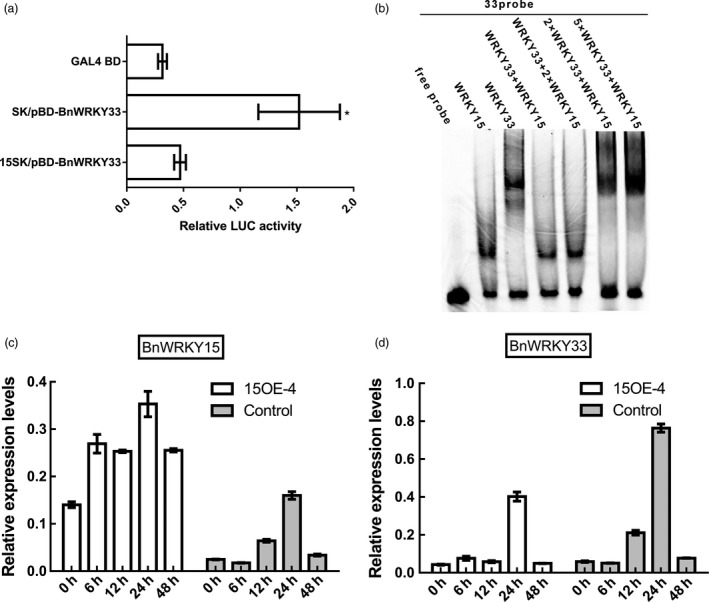
*BnWRKY15*‐mediated modulation of *BnWRKY33*. (a) Transcriptional activation ability changes of BnWRKY33 were revealed by the relative LUC activity of the reporter. *Arabidopsis* protoplasts were cotransformed with pBD‐BnWRKY33 effector plasmids (Figure 7a) and 15SK plasmids (Figure 4a) together with reporter plasmids. Transcriptional activation ability produced by cotransforming the null plasmid pGreenII 62‐SK (Figure 4a) with the pBD‐BnWRKY33 effector into *Arabidopsis* protoplasts was used as a control. The GAL4 BD effector served as a negative control. The assays were repeated at least three times. The data represent the means ± standard errors (*n* ≥ 3). (b) Competitive binding of BnWRKY15 and BnWRKY33 to the promoter of *BnWRKY33*. Electrophoretic mobility shift assays were performed using recombinant BnWRKY15 or BnWRKY33 proteins and Cy5‐labelled W‐box probes. Equal amounts of recombinant BnWRKY15 and BnWRKY33 protein or a 2:1 ratio of BnWRKY15:BnWRKY33 fusion protein was incubated with Cy5‐labelled W‐box probes and separated using nondenaturing polyacrylamide gel electrophoresis. W‐box probes alone were used as controls. (c) Expression levels of *BnWRKY15* in both the *BnWRKY15‐*overexpressing line and the control (Westar) after inoculation with *Sclerotinia sclerotiorum*. (d) Expression levels of *BnWRKY33* in both the *BnWRKY15‐*overexpressing line and the control (Westar) after inoculation with *S*.* sclerotiorum*. Columns with different shades of grey indicate expression levels at different time points. Quantitative results represent three biological repeats. The error bars stand for standard error.

To investigate the mechanism by which *BnWRKY15* represses *BnWRKY33* expression, as was observed in the transgenic plants and *Arabidopsis* protoplast assays (Figures 7 and 11), we examined the effects of *BnWRKY15* on the transcriptional activation of *BnWRKY33*. The reporter gene expression caused by the pBD‐BnWRKY33 effector (Figure 9a) decreased by approximately 30% in the presence of the *BnWRKY15*‐overexpressing plasmid (15SK; Figure 7a) compared with that of the control cotransformed with the pGreenII 62‐SK plasmid (SK; Figure 12a).

EMSA was subsequently used to evaluate the competitive binding abilities of BnWRKY15 and BnWRKY33 to the W‐box *in vitro*. The results showed that recombinant BnWRKY15 and BnWRKY33 proteins could individually bind to the W‐box of the *BnWRKY33* promoter region (Figure 12b). However, when equal amounts of BnWRKY15 and BnWRKY33 protein were incubated together with the W‐box probes, only the BnWRKY15 protein could bind to the probe; when a 2:1 ratio of BnWRKY15:BnWRKY33 was used, the effect was more pronounced (Figure 12b). However, the lagging band from BnWRKY33 was only observed when the BnWRKY33 fusion protein was added at amounts twofold or fivefold greater than the amount of the BnWRKY15 fusion protein (Figure 12b).

Furthermore, qPCR quantification of *BnWRKY15* and *BnWRKY33* expression in the *BnWRKY15*‐overexpressing and control lines, whose infections with *S. sclerotiorum* persisted for different times, indicated that *BnWRKY15* expression was higher in the overexpressing line than in the control and that the expression of *BnWRKY33* was suppressed to a similar level (except at the 24‐h time point) (Figure 12c and d).

## Discussion

### Interaction between BnWRKY15 and BnWRKY33

The DNA‐binding domain of WRKY proteins (WRKY domain) is the defining feature of WRKY TFs (Rushton *et al*., 2010), as this domain binds to W‐box elements in the promoters of target genes to activate or repress their expression. Interactions between WRKY proteins and their targets are well demonstrated (Rushton *et al*., 2010). Moreover, W‐box elements in the promoters of WRKY TFs are targeted by other WRKY TFs, and some WRKY TFs are self‐regulating (Chen *et al*., 2010; Eulgem *et al*., 1999; Mao *et al*., 2011; Yan *et al*., 2012). Our finding that *BnWRKY33* expression is regulated by both *BnWRKY15* and itself provides additional support for this phenomenon.

The regulation of a TF can occur in two different ways: passive repression and active repression (Gaston and Jayaraman, 2003). Active repression processes inhibit the initiation of transcription directly via the actions of independent repression domains (Hanna‐Rose and Hansen, 1996). In the present study, activation and repression domains were found to clearly exist in the two BnWRKYs. Moreover, BnWRKY15 exhibited weak transcriptional repression and could also reduce the transactivation ability of *BnWRKY33*, which indicates that this protein has features similar to those of the class II apetala2/ethylene response factors (AP2/ERFs) and Cys2/His2‐type zinc‐finger proteins that contain an EAR motif, that is the ability to repress the transactivation of reporter genes and other TFs (Ohta *et al*., 2001). Therefore, the repression mechanism of *BnWRKY15* might be attributed to the reduced transcriptional activation ability of *BnWRKY33*. In addition, the EMSA results suggested that BnWRKY15 had a higher affinity for the W‐box than did BnWRKY33, which is in accordance with passive TF regulation; however, further *in vivo* evidence might be needed for confirmation.

Although *BnWRKY15* could regulate the expression of *BnWRKY33*, MPK3/MPK6‐activated BnWRKY33 (Mao *et al*., 2011) could have stronger transactivation ability compared with that of BnWRKY15 and could activate the expression of *BnWRKY33* under the control of itself after treatment with *S. sclerotiorum*. These phenomena might explain why both *BnWRKY*s simultaneously increase after the *S. sclerotiorum* treatment. The positive feedback regulation loop formed by AtWRKY33 that enhances camalexin synthesis in response to *B*. *cinerea* was proposed in *Arabidopsis* by Mao *et al*. (2011). Similarly, considering that BnWRKY33 could also activate its own expression, we assumed that the positive regulatory loop mediated by *BnWRKY33* also occurs in *B. napus*. Indeed, the activation of *LUC* expression by *BnWRKY33* was detected in the absence of an elicitor such as *S. sclerotiorum*. Accordingly, we concluded that BnWRKY33 might activate its own expression by binding to the W‐box, even without infection by *S. sclerotiorum*. Thus, another factor such as *BnWRKY15* might be needed to regulate the excessive expression of *BnWRKY33* transcripts in the absence of *S. sclerotiorum* infection.

*WRKY33* expression and camalexin synthesis are induced in the *Atmpk3/Atmpk6* double mutant (Mao *et al*., 2011), which might indicate that another pathway modulates the expression of *BnWRKY33* and subsequently activates camalexin synthesis. However, in the present study, WRKY15 could bind to the W‐box in the *WRKY33* promoter and activate *WRKY33* expression. This finding implicates *WRKY15* as an activator of *WRKY33* expression in the *Atmpk3/Atmpk6* double mutant.

Although the repression function of *BnWRKY15* was shown in *Arabidopsis* protoplasts and in *BnWRKY15*‐overexpressing plants, the negative regulation was incomplete, as *BnWRKY15* retained some transcriptional initiation activity when co‐expressed with P‐346‐LUC constructs. Therefore, we speculate that this phenomenon might be explained from two aspects: first, the transactivation in co‐expression of 15SK with P‐346‐LUC constructs was from comparing with LUC activity of co‐expression 15SK with constructs lacking W1 region (P‐W2W3‐LUC, P‐W3‐LUC, P‐249‐LUC), but not by comparing with LUC activity from only expression of P‐346‐LUC constructs; and second, BnWRKY15 could own transactivation domain from the activation or repression domain assay, and then, BnWRKY15 might play the role as transcriptional activator at some cases. If BnWRKY15 could act as transactivator of BnWRKY33, then the activated transcription of *BnWRKY33* caused by *BnWRKY15* should be weaker than the influence caused by the repression of the transactivation ability of *BnWRKY33* by *BnWRKY15*, because the co‐expression of both *BnWRKY*s with P‐346‐LUC constructs showed a similar reporter expression as did the transfection of BnWRKY15 alone with P‐346‐LUC constructs. Additionally, down‐regulation of *BnWRKY33* in BnWRKY15‐overexpressing plants could also be attributed to this phenomenon.

Plant defence systems carry fitness costs and require the allocation of limited resources that could otherwise be used for growth or reproduction (Bostock, 2005). Therefore, such defence systems need to be tightly and finely regulated. In addition to being an enormous waste of energy, constitutively activated defence responses can cause hypersensitive responses and even stunted growth and low fertility (Lorrain *et al*., 2003). Thus, appropriate regulatory factors such as *BnWRKY15* are necessary to modulate defences at a low level or to prevent the activation of defences in order to balance resource allocation.

In fact, W‐box clusters occur in the WRKY‐binding regions of target gene promoters (Chen and Chen, 2002; Du and Chen, 2000; Lippok *et al*., 2007; Wang *et al*., 2009). Indeed, a cluster of three W‐box elements was identified in the promoter of *BnWRKY33*, and all three boxes could be bound by WRKYs, although only binding of the first W‐box resulted in *BnWRKY33* activation. However, the activation of the two WRKYs was abolished when the three native W‐box elements were replaced with four W1‐box elements. Thus, the W‐box cluster might cooperate in the activation of transcription, as indicated previously (Eulgem *et al*., 1999). In addition, the P‐W2W3 reporter more severely reduced *LUC* expression than did P‐W1 m5, which suggests that the nucleotide bases neighbouring the W1‐box also contribute to the binding of WRKY TFs.

### Roles of *BnWRKY15* and *BnWRKY33* in response to *S. sclerotiorum*

*BnWRKY15* and its homolog *AtWRKY15* belong to the group IId WRKY TFs (Eulgem *et al*., 2000). According to previous reports, most members of the group IId WRKY TFs negatively regulate the responses of plants to biotic and abiotic stresses (Journot‐Catalino *et al*., 2006; Kim *et al*., 2006; Vanderauwera *et al*., 2012). *AtWRKY15* is reported to play a role in modulating plant growth and salt/osmotic stress responses (Vanderauwera *et al*., 2012); however, in the present study, our results showed that *BnWRKY15* overexpression compromised the resistance of *B. napus* to *S. sclerotiorum*. In addition, the expression of *BnWRKY33*, which was reported to confer *S. sclerotiorum* resistance by Wang *et al*. (2014) and in this present study, was repressed by *BnWRKY15* overexpression. Similarly, the expression of *BnPAD3* and *BnCYP71A13* decreased in *BnWRKY15*‐overexpressing plants. Thus, the moderate transcriptional repression of *BnWRKY33* by *BnWRKY15* might be responsible for the lower expression of *BnWRKY33* compared with that in the control; therefore, reduced *BnWRKY33* expression might contribute to the lower expression of *BnPAD3* and *BnCYP71A13*, which would explain the susceptibility of *BnWRKY15*‐overexpressing plants to infection.

*AtWRKY33* participates in plant resistance to necrotrophic pathogens (Zheng *et al*., 2006). In the present study, the similarly induced homolog of *AtWRKY33*,* BnWRKY33* (Lippok *et al*., 2007), enhanced the resistance of *B. napus* to *S. sclerotiorum* in the overexpressing lines. Moreover, the elevated expression of the SA‐ and JA‐regulated defence responses genes in *BnWRKY33*‐overexpressing plants suggests that *BnWRKY33* overexpression activates both SA and JA signalling, which were recently shown to be involved in the defence response of *B. napus* to *S. sclerotiorum* (Nováková *et al*., 2014; Wang *et al*., 2012). Therefore, the activation of these signalling pathways might explain the resistance‐inducing properties of *BnWRKY33*.

Camalexin plays an important role in plant responses to various pathogens, including *S. sclerotiorum* (Ferrari *et al*., 2003; Glawischnig, 2007; Nafisi *et al*., 2007; Schuhegger *et al*., 2006; Stotz *et al*., 2011; Zhou *et al*., 1999). In the present study, the elevated expression of *PAD3* and *CYP71A13* in transgenic plants and the transactivator feature of BnWRKY33 suggest that *BnWRKY33* functions as a typical TF and may activate downstream genes, such as *PAD3* and *CYP71A13*. Thereby, it may be concluded that *BnWRKY33* overexpression could enhance the transcription of *PAD3* and *CYP71A13*, subsequently participating in the synthesis of camalexin and enhancing resistance to *S. sclerotiorum*.

## Experimental procedures

### cDNA synthesis and qPCR analysis

Total RNA was isolated using an RNApure High‐Purity Total RNA Rapid Extraction Kit (Bio‐Tech), and first‐strand cDNA was synthesized from 1 μg of total RNA in 20‐μL reactions using a First Strand cDNA Synthesis Kit (Fermentas) and oligo‐dT(18)‐MN primers in accordance with the manufacturer's instructions. Sequences of *B. napus* genes used for qPCR were identified using BLASTN to search for homologs of corresponding gene sequences from *A. thaliana*. Primers were designed using PrimerExpress3.0 (Applied Biosystems). qPCR was performed using SYBR Green Real‐Time PCR Master Mix (Toyobo) under the following conditions: polymerase activation for 2 min at 95 °C, followed by 40 cycles of 15 s at 95 °C, 30 s at 60 °C and 30 s at 72 °C. A melting curve was constructed by performing 60 cycles of 5 s at 65 °C in conjunction with a 0.5 °C increase in temperature for each cycle. The *B. napus Actin* (*BnActin*, AF111812.1) gene was used as reference for internal control. All the qPCR data were presented as the relative quantification [2^(−ΔCT)^] between target genes and the *Actin* gene.

### Generation of transgenic *B. napus*

To develop *BnWRKY33*‐overexpressing plants, the full‐length open reading frame (ORF) of *BnWRKY33* was obtained from the NCBI database using BLASTN and sequences from the suppression subtractive hybridization library and then amplified from the cultivar Ning RS‐1 (Table S2). The obtained sequence was then used to design IP molecular marker 33‐56yh, which showed differential amplification between parent lines and individuals of the TN population. In addition, the TN DH population was used for mapping *BnWRKY33*. The ORF of *BnWRKY33* was then inserted into the pBI121 vector (Jefferson *et al*., 1987) to generate a construct that consisted of *BnWRKY33* under the control of a double CaMV 35S promoter. In contrast, a construct with BnWRKY15 under the control of a CaMV 35S promoter was generated by amplifying and cloning the *BnWRKY15* ORF into the pBI121s vector, which was constructed from pBI121, using *Eco*RI and *Hind*III to insert a fragment that contained both a double 35S promoter from the CaMV and a terminator from the pCAMBIA1300s plasmid (Xiong and Yang, 2003). The constructs were then transferred into *Agrobacterium tumefaciens* GV3101 and were subsequently used to transform *B. napus* (Westar) as described previously (De Block *et al*., 1989).

### Promoter analysis

The *BnWRKY33* promoter sequence was identified using thermal asymmetric interlaced PCR (TAIL‐PCR) (Singer and Burke, 2003), and the promoter sequence of *BnWRKY15* was identified using BLASTN to query the *B. napus* genome (Chalhoub *et al*., 2014). The obtained *BnWRKY33* promoter sequence was analysed using PlantPAN2.0 software. Constructs for promoter analysis were prepared by amplifying relevant *BnWRKY33* promoter sequences containing 5′ ATG upstream regions 1000 (P‐33), 346 (P‐346) and 249 bp (P‐249) in length using PCR (Table S2) and by introducing into these sequences into pBI101 to generate the P‐33‐GUS, P‐346‐GUS and P‐249‐GUS constructs, respectively. On the other hand, the *BnWRKY15* promoter sequence containing a 5′ ATG upstream promoter region of 1225 bp (P‐15) was used to construct a P‐15‐GUS reporter. The constructs were then transformed into *Arabidopsis* in accordance with the floral dip method (Clough and Bent, 1998), and GUS staining was performed as described previously (Willemsen *et al*., 1998). For the analysis of *S. sclerotiorum‐*, SA‐ and H_2_O_2_‐induced GUS activity, *Arabidopsis* rosette leaves were treated and harvested as described above.

### Yeast one‐hybrid assays

To screen upstream TFs for *BnWRKY33* promoter‐binding capability, a yeast one‐hybrid assay was performed using the Matchmaker Gold Yeast One‐Hybrid System (Clontech) in accordance with the manufacturer's instructions. Briefly, the bait plasmid 33box‐pAbAi was constructed by inserting the 33box fragment of the *BnWRKY33* promoter in front of the *AUR1‐C* gene, which is an antibiotic resistance gene in the pAbAi plasmid that confers resistance to AbA. In addition, reporter strains were generated by integrating linearized 33box‐pAbAi or null pAbAi plasmids into the genome of the yeast strain Y1HGold, and the appropriate inhibition concentration of AbA to the bait reporter strains was confirmed according to the manufacturer's instructions. RNA from *B. napus* (Ning RS‐1) leaves harvested at 24 and 48 h after *S. sclerotiorum* inoculation was used for reverse transcription, and the obtained cDNA was fused with the GAL4 activation domain of pGADT7‐REC to construct library for yeast one‐hybrid screen assay. The protein–DNA interaction is identified by activation of the AbA resistance gene when a prey protein from the library binds to the bait sequence. The library was screened on SD/‐Leu medium that contained 100 ng/mL AbA. The plasmid pGADT7‐Rec‐BnWRKY15 was rescued from positive yeast colonies and retransformed into bait reporter strains for interaction validation.

### EMSA

Full‐length *BnWRKY15* and *BnWRKY33* cDNAs were cloned into the pET‐32a expression vector (Novagen) and transferred into the *Escherichia coli* strain Rosetta (DE3). Recombinant protein expression was induced using isopropyl β‐D‐1‐thiogalactopyranoside (0.25 mm for BnWRKY33 or 0.5 mm for BnWRKY15), and the proteins were purified in accordance with the manufacturer's (Novagen) instructions. Two complementary oligonucleotide strands were labelled with Cy5 and annealed to generate probes, and the purified recombinant proteins were incubated in binding buffer (Beyotime) at room temperature in the presence of 40 nm DNA probe and in the presence or absence of unlabelled competitor DNA. Finally, the DNA–protein complexes were electrophoresed on 6% nondenaturing polyacrylamide gels in an ice water bath.

### Construction of plasmids for *Arabidopsis* protoplast transient assays

To verify the binding of BnWRKY15 or BnWRKY33 to the W‐box *in vivo*, we performed transient dual‐luciferase reporter assays as described previously (Hellens *et al*., 2005). The P‐346 region (−346 to −1 bp), which included all three W‐box elements; the P‐W2W3 region (−314 to −1 bp), in which the first W‐box was deleted; the P‐W3 region (−297 to −1 bp), in which the first and second W‐box elements were deleted; and the P‐249 region (−249 to −1 bp), in which all three W‐box elements were deleted, were individually cloned into the pGreenII 0800‐LUC reporter plasmid to generate P‐346‐LUC, P‐W2W3‐LUC, P‐W3‐LUC and P‐249‐LUC, respectively. We also fused the *LUC* gene to five versions of the P‐346 region containing different mutations in the W1 region as well as to a mutant in which W1, W2 and W3 were replaced with four W1 regions to generate P‐W1m1‐LUC, P‐W1m2‐LUC, P‐W1m3‐LUC, P‐W1m4‐LUC, P‐W1m5‐LUC and P‐4W1‐LUC, respectively. Furthermore, coding regions of *BnWRKY15* and *BnWRKY33* were inserted into pGreenII 62‐SK to generate the effectors 15SK and 33SK.

In addition, to assess the transcriptional activation of *BnWRKY15* and *BnWRKY33*, we constructed GAL4 reporter plasmids by inserting the LUC gene driven by the minimal TATA box of the 35S promoter plus five GAL4‐binding elements into pUC19 (Ohta *et al*., 2001). ORFs of *BnWRKY15* and *BnWRKY33* (Table S2) were fused with the GAL4‐binding domain to generate pBD‐BnWRKY33 and pBD‐BnWRKY15 constructs. The *Renilla* luciferase gene driven by the *Arabidopsis* ubiquitin (UBQ3) promoter was used as an internal control. To characterize the activation or repression domains of BnWRKY15 and BnWRKY33, we fused different deletions of the two BnWRKYs (shown in Figure S9) to the GAL4‐binding domain to generate different deletion effectors. Isolation and transformation of *Arabidopsis* protoplasts were performed as described in Data S1.

Plant growth and treatment, resistance assay and subcellular protein localization are placed in Data S1.

All the sequences of primers and probes are shown in Table S2.

## Supporting information


**Figure S1** Map‐based location of *BnWRKY33* in the A05 linkage group.
**Figure S2** Expression levels of partial *BnWRKY33*‐overexpressing lines (T_0_ generation).
**Figure S3** *Cis*‐elements identified using the *BnWRKY33* promoter from rice and the PlantPAN2.0 software.
**Figure S4** β‐Glucuronidase (GUS) staining of *Arabidopsis* plants containing P‐346‐GUS or P‐249‐GUS constructs.
**Figure S5** Amino acid comparison of BnWRKY15 and its homologous protein in *Arabidopsis*, AtWRKY15.
**Figure S6** Transcriptional activation of truncated *BnWRKY15* and *BnWRKY33* genes in *Arabidopsis* protoplasts.
**Figure S7** Subcellular localization of the *BnWRKY15* and *BnWRKY33* genes.
**Figure S8** β‐Glucuronidase (GUS) histochemical staining and activities of transgenic *Arabidopsis* plants harbouring P‐15‐GUS.
**Figure S9** Expression levels of *BnWRKY15*‐overexpressing lines (T_0_ generation).
**Table S1** Lesion areas of both *BnWRKY33*‐overexpressing and control plants at 48 h (T_0_) after inoculation with *Sclerotinia sclerotiorum*.
**Table S2** Primers used for cloning, plasmid construction and quantitative RT‐PCR.
**Table S3** Annotation of colonies detected using yeast one‐hybrid assays.

**Data S1** Methods.

## References

[pbi12838-bib-0001] Andreasson, E. , Jenkins, T. , Brodersen, P. , Thorgrimsen, S. , Petersen, N.H. , Zhu, S. , Qiu, J.L. *et al* (2005) The MAP kinase substrate MKS1 is a regulator of plant defense responses. EMBO J. 24, 2579–2589. 15990873 10.1038/sj.emboj.7600737 PMC1176463

[pbi12838-bib-0002] Boland, G.J. and Hall, R. (1994) Index of plant hosts of Sclerotinia sclerotiorum. Can. J. Plant Path. 16, 93–108.

[pbi12838-bib-0003] Bostock, R.M. (2005) Signal crosstalk and induced resistance: straddling the line between cost and benefit. Annu. Rev. Phytopathol. 43, 545–580. 16078895 10.1146/annurev.phyto.41.052002.095505

[pbi12838-bib-0004] Chalhoub, B. , Denoeud, F. , Liu, S. , Parkin, I.A. , Tang, H. , Wang, X. , Chiquet, J. *et al* (2014) Plant genetics. Early allopolyploid evolution in the post‐Neolithic Brassica napus oilseed genome. Science, 345, 950–953. 25146293 10.1126/science.1253435

[pbi12838-bib-0005] Chen, C. and Chen, Z. (2002) Potentiation of developmentally regulated plant defense response by AtWRKY18, a pathogen‐induced Arabidopsis transcription factor. Plant Physiol. 129, 706–716. 12068113 10.1104/pp.001057 PMC161695

[pbi12838-bib-0006] Chen, H. , Lai, Z. , Shi, J. , Xiao, Y. , Chen, Z. and Xu, X. (2010) Roles of arabidopsis WRKY18, WRKY40 and WRKY60 transcription factors in plant responses to abscisic acid and abiotic stress. BMC Plant Biol. 10, 281. 21167067 10.1186/1471-2229-10-281 PMC3023790

[pbi12838-bib-0007] Cheng, Y. , Zhou, Y. , Yang, Y. , Chi, Y.‐J. , Zhou, J. , Chen, J.‐Y. , Wang, F. *et al* (2012) Structural and functional analysis of VQ motif‐containing proteins in Arabidopsis as interacting proteins of WRKY transcription factors. Plant Physiol. 159, 810–825. 22535423 10.1104/pp.112.196816 PMC3375943

[pbi12838-bib-0008] Clough, S.J. and Bent, A.F. (1998) Floral dip: a simplified method for Agrobacterium‐mediated transformation of Arabidopsis thaliana. Plant J. 16, 735–743. 10069079 10.1046/j.1365-313x.1998.00343.x

[pbi12838-bib-0009] Dang, F.F. , Wang, Y.N. , Yu, L. , Eulgem, T. , Lai, Y. , Liu, Z.Q. , Wang, X. *et al* (2013) CaWRKY40, a WRKY protein of pepper, plays an important role in the regulation of tolerance to heat stress and resistance to Ralstonia solanacearum infection. Plant, Cell Environ. 36, 757–774. 22994555 10.1111/pce.12011

[pbi12838-bib-0010] De Block, M. , De Brouwer, D. and Tenning, P. (1989) Transformation of Brassica napus and Brassica oleracea using Agrobacterium tumefaciens and the expression of the bar and neo Genes in the transgenic plants. Plant Physiol. 91, 694–701. 16667089 10.1104/pp.91.2.694 PMC1062058

[pbi12838-bib-0011] Du, L. and Chen, Z. (2000) Identification of genes encoding receptor‐like protein kinases as possible targets of pathogen‐and salicylic acid‐induced WRKY DNA‐binding proteins in Arabidopsis. Plant J. 24, 837–847. 11135117 10.1046/j.1365-313x.2000.00923.x

[pbi12838-bib-0012] Eulgem, T. and Somssich, I.E. (2007) Networks of WRKY transcription factors in defense signaling. Curr. Opin. Plant Biol. 10, 366–371. 17644023 10.1016/j.pbi.2007.04.020

[pbi12838-bib-0013] Eulgem, T. , Rushton, P.J. , Schmelzer, E. , Hahlbrock, K. and Somssich, I.E. (1999) Early nuclear events in plant defence signalling: rapid gene activation by WRKY transcription factors. EMBO J. 18, 4689–4699. 10469648 10.1093/emboj/18.17.4689 PMC1171542

[pbi12838-bib-0014] Eulgem, T. , Rushton, P.J. , Robatzek, S. and Somssich, I.E. (2000) The WRKY superfamily of plant transcription factors. Trends Plant Sci. 5, 199–206. 10785665 10.1016/s1360-1385(00)01600-9

[pbi12838-bib-0015] Ferrari, S. , Plotnikova, J.M. , De Lorenzo, G. and Ausubel, F.M. (2003) Arabidopsis local resistance to Botrytis cinerea involves salicylic acid and camalexin and requires EDS4 and PAD2, but not SID2, EDS5 or PAD4. Plant J. 35, 193–205. 12848825 10.1046/j.1365-313x.2003.01794.x

[pbi12838-bib-0016] Garg, H. , Li, H. , Sivasithamparam, K. and Barbetti, M.J. (2013) Differentially expressed proteins and associated histological and disease progression changes in cotyledon tissue of a resistant and susceptible genotype of Brassica napus infected with Sclerotinia sclerotiorum. PLoS ONE, 8, e65205. 23776450 10.1371/journal.pone.0065205 PMC3679123

[pbi12838-bib-0017] Gaston, K. and Jayaraman, P.S. (2003) Transcriptional repression in eukaryotes: repressors and repression mechanisms. Cellular Molecular Life Sci. 60, 721–741. 10.1007/s00018-003-2260-3 PMC11138846 12785719

[pbi12838-bib-0018] Glawischnig, E. (2007) Camalexin. Phytochemistry, 68, 401–406. 17217970 10.1016/j.phytochem.2006.12.005

[pbi12838-bib-0019] Hanna‐Rose, W. and Hansen, U. (1996) Active repression mechanisms of eukaryotic transcription repressors. Trends Genet. 12, 229–234. 8928228 10.1016/0168-9525(96)10022-6

[pbi12838-bib-0020] Hellens, R.P. , Allan, A.C. , Friel, E.N. , Bolitho, K. , Grafton, K. , Templeton, M.D. , Karunairetnam, S. *et al* (2005) Transient expression vectors for functional genomics, quantification of promoter activity and RNA silencing in plants. Plant Methods, 1, 13. 16359558 10.1186/1746-4811-1-13 PMC1334188

[pbi12838-bib-0021] Jefferson, R.A. , Kavanagh, T.A. and Bevan, M.W. (1987) GUS fusions: beta‐glucuronidase as a sensitive and versatile gene fusion marker in higher plants. EMBO J. 6, 3901. 3327686 10.1002/j.1460-2075.1987.tb02730.x PMC553867

[pbi12838-bib-0022] Journot‐Catalino, N. , Somssich, I.E. , Roby, D. and Kroj, T. (2006) The transcription factors WRKY11 and WRKY17 act as negative regulators of basal resistance in Arabidopsis thaliana. Plant Cell Online, 18, 3289–3302. 10.1105/tpc.106.044149 PMC1693958 17114354

[pbi12838-bib-0023] Kim, K.‐C. , Fan, B. and Chen, Z. (2006) Pathogen‐induced Arabidopsis WRKY7 is a transcriptional repressor and enhances plant susceptibility to Pseudomonas syringae. Plant Physiol. 142, 1180–1192. 16963526 10.1104/pp.106.082487 PMC1630724

[pbi12838-bib-0024] Lagercrantz, U. (1998) Comparative mapping between Arabidopsis thaliana and Brassica nigra indicates that Brassica genomes have evolved through extensive genome replication accompanied by chromosome fusions and frequent rearrangements. Genetics, 150, 1217–1228. 9799273 10.1093/genetics/150.3.1217 PMC1460378

[pbi12838-bib-0025] Li, S. , Zhou, X. , Chen, L. , Huang, W. and Yu, D. (2010) Functional characterization of Arabidopsis thaliana WRKY39 in heat stress. Mol. Cells, 29, 475–483. 20396965 10.1007/s10059-010-0059-2

[pbi12838-bib-0026] Liang, Y. , Srivastava, S. , Rahman, M.H. , Strelkov, S.E. and Kav, N.N. (2008) Proteome changes in leaves of Brassica napus L. as a result of Sclerotinia sclerotiorum challenge. J. Agric. Food Chem. 56, 1963–1976. 18290614 10.1021/jf073012d

[pbi12838-bib-0027] Lippok, B. , Birkenbihl, R.P. , Rivory, G. , Brummer, J. , Schmelzer, E. , Logemann, E. and Somssich, I.E. (2007) Expression of AtWRKY33 encoding a pathogen‐ or PAMP‐responsive WRKY transcription factor is regulated by a composite DNA motif containing W box elements. Molecular Plant‐Microbe Interact. 20, 420–429. 10.1094/MPMI-20-4-0420 17427812

[pbi12838-bib-0028] Liu, S. , Kracher, B. , Ziegler, J. , Birkenbihl, R.P. and Somssich, I.E. (2015) Negative regulation of ABA signaling by WRKY33 is critical for Arabidopsis immunity towards Botrytis cinerea 2100. Elife, 4, e07295. 26076231 10.7554/eLife.07295 PMC4487144

[pbi12838-bib-0029] Lorrain, S. , Vailleau, F. , Balague, C. and Roby, D. (2003) Lesion mimic mutants: keys for deciphering cell death and defense pathways in plants? Trends Plant Sci. 8, 263–271. 12818660 10.1016/S1360-1385(03)00108-0

[pbi12838-bib-0030] Luo, M. , Dennis, E.S. , Berger, F. , Peacock, W.J. and Chaudhury, A. (2005) MINISEED3 (MINI3), a WRKY family gene, and HAIKU2 (IKU2), a leucine‐rich repeat (LRR) KINASE gene, are regulators of seed size in Arabidopsis. Proc. Natl Acad. Sci. USA, 102, 17531–17536. 16293693 10.1073/pnas.0508418102 PMC1297679

[pbi12838-bib-0031] Mao, G. , Meng, X. , Liu, Y. , Zheng, Z. , Chen, Z. and Zhang, S. (2011) Phosphorylation of a WRKY transcription factor by two pathogen‐responsive MAPKs drives phytoalexin biosynthesis in Arabidopsis. Plant Cell, 23, 1639–1653. 21498677 10.1105/tpc.111.084996 PMC3101563

[pbi12838-bib-0032] Meng, X. and Zhang, S. (2013) MAPK cascades in plant disease resistance signaling. Annu. Rev. Phytopathol. 51, 245–266. 23663002 10.1146/annurev-phyto-082712-102314

[pbi12838-bib-0033] Nafisi, M. , Goregaoker, S. , Botanga, C.J. , Glawischnig, E. , Olsen, C.E. , Halkier, B.A. and Glazebrook, J. (2007) Arabidopsis cytochrome P450 monooxygenase 71A13 catalyzes the conversion of indole‐3‐acetaldoxime in camalexin synthesis. Plant Cell, 19, 2039–2052. 17573535 10.1105/tpc.107.051383 PMC1955726

[pbi12838-bib-0034] Nováková, M. , Šašek, V. , Dobrev, P.I. , Valentová, O. and Burketová, L. (2014) Plant hormones in defense response of Brassica napus to Sclerotinia sclerotiorum–Reassessing the role of salicylic acid in the interaction with a necrotroph. Plant Physiol. Biochem. 80, 308–317. 24837830 10.1016/j.plaphy.2014.04.019

[pbi12838-bib-0035] Ohta, M. , Matsui, K. , Hiratsu, K. , Shinshi, H. and Ohme‐Takagi, M. (2001) Repression domains of class II ERF transcriptional repressors share an essential motif for active repression. Plant Cell, 13, 1959–1968. 11487705 10.1105/TPC.010127 PMC139139

[pbi12838-bib-0036] Pandey, S.P. and Somssich, I.E. (2009) The role of WRKY transcription factors in plant immunity. Plant Physiol. 150, 1648–1655. 19420325 10.1104/pp.109.138990 PMC2719123

[pbi12838-bib-0037] Qiu, D. , Morgan, C. , Shi, J. , Long, Y. , Liu, J. , Li, R. , Zhuang, X. *et al* (2006) A comparative linkage map of oilseed rape and its use for QTL analysis of seed oil and erucic acid content. Theoret. Appl. Genet. 114, 67–80. 17033785 10.1007/s00122-006-0411-2

[pbi12838-bib-0038] Qiu, D. , Xiao, J. , Ding, X. , Xiong, M. , Cai, M. , Cao, Y. , Li, X. *et al* (2007) OsWRKY13 mediates rice disease resistance by regulating defense‐related genes in salicylate‐and jasmonate‐dependent signaling. Mol. Plant Microbe Interact. 20, 492–499. 17506327 10.1094/MPMI-20-5-0492

[pbi12838-bib-0039] Qiu, J.L. , Fiil, B.K. , Petersen, K. , Nielsen, H.B. , Botanga, C.J. , Thorgrimsen, S. , Palma, K. *et al* (2008) Arabidopsis MAP kinase 4 regulates gene expression through transcription factor release in the nucleus. EMBO J. 27, 2214–2221. 18650934 10.1038/emboj.2008.147 PMC2519101

[pbi12838-bib-0040] Rushton, P.J. , Somssich, I.E. , Ringler, P. and Shen, Q.J. (2010) WRKY transcription factors. Trends Plant Sci. 15, 247–258. 20304701 10.1016/j.tplants.2010.02.006

[pbi12838-bib-0041] Schmidt, R. , Acarkan, A. and Boivin, K. (2001) Comparative structural genomics in the Brassicaceae family. Plant Physiol. Biochem. 39, 253–262.

[pbi12838-bib-0042] Schuhegger, R. , Nafisi, M. , Mansourova, M. , Petersen, B.L. , Olsen, C.E. , Svatos, A. , Halkier, B.A. *et al* (2006) CYP71B15 (PAD3) catalyzes the final step in camalexin biosynthesis. Plant Physiol. 141, 1248–1254. 16766671 10.1104/pp.106.082024 PMC1533948

[pbi12838-bib-0043] Singer, T. and Burke, E. (2003) High‐throughput TAIL‐PCR as a tool to identify DNA flanking insertions In Plant Functional Genomics (Grotewold E., ed), pp. 241–271. Totowa, New Jersey: Springer. 10.1385/1-59259-413-1:241 14501069

[pbi12838-bib-0044] Singh, K. , Foley, R.C. and Onate‐Sanchez, L. (2002) Transcription factors in plant defense and stress responses. Curr. Opin. Plant Biol. 5, 430–436. 12183182 10.1016/s1369-5266(02)00289-3

[pbi12838-bib-0045] Stotz, H.U. , Sawada, Y. , Shimada, Y. , Hirai, M.Y. , Sasaki, E. , Krischke, M. , Brown, P.D. *et al* (2011) Role of camalexin, indole glucosinolates, and side chain modification of glucosinolate‐derived isothiocyanates in defense of Arabidopsis against Sclerotinia sclerotiorum. Plant J. 67, 81–93. 21418358 10.1111/j.1365-313X.2011.04578.x

[pbi12838-bib-0046] Tao, Z. , Liu, H. , Qiu, D. , Zhou, Y. , Li, X. , Xu, C. and Wang, S. (2009) A pair of allelic WRKY genes play opposite roles in rice‐bacteria interactions. Plant Physiol. 151, 936–948. 19700558 10.1104/pp.109.145623 PMC2754648

[pbi12838-bib-0047] Turck, F. , Zhou, A. and Somssich, I.E. (2004) Stimulus‐dependent, promoter‐specific binding of transcription factor WRKY1 to its native promoter and the defense‐related gene PcPR1‐1 in parsley. Plant Cell, 16, 2573–2585. 15367720 10.1105/tpc.104.024810 PMC520956

[pbi12838-bib-0048] Ulker, B. and Somssich, I.E. (2004) WRKY transcription factors: from DNA binding towards biological function. Curr. Opin. Plant Biol. 7, 491–498. 15337090 10.1016/j.pbi.2004.07.012

[pbi12838-bib-0049] Vanderauwera, S. , Vandenbroucke, K. , Inze, A. , van de Cotte, B. , Muhlenbock, P. , De Rycke, R. , Naouar, N. *et al* (2012) AtWRKY15 perturbation abolishes the mitochondrial stress response that steers osmotic stress tolerance in Arabidopsis. Proc. Natl Acad. Sci. USA, 109, 20113–20118. 23169634 10.1073/pnas.1217516109 PMC3523852

[pbi12838-bib-0050] Wang, Z. , Zhu, Y. , Wang, L. , Liu, X. , Liu, Y. , Phillips, J. and Deng, X. (2009) A WRKY transcription factor participates in dehydration tolerance in Boea hygrometrica by binding to the W‐box elements of the galactinol synthase (BhGolS1) promoter. Planta, 230, 1155–1166. 19760263 10.1007/s00425-009-1014-3

[pbi12838-bib-0051] Wang, J. , Lydiate, D.J. , Parkin, I.A. , Falentin, C. , Delourme, R. , Carion, P.W. and King, G.J. (2011) Integration of linkage maps for the Amphidiploid Brassica napus and comparative mapping with Arabidopsis and Brassica rapa. BMC Genom. 12, 101. 10.1186/1471-2164-12-101 PMC3042011 21306613

[pbi12838-bib-0052] Wang, Z. , Tan, X. , Zhang, Z. , Gu, S. , Li, G. and Shi, H. (2012) Defense to Sclerotinia sclerotiorum in oilseed rape is associated with the sequential activations of salicylic acid signaling and jasmonic acid signaling. Plant Sci. 184, 75–82. 22284712 10.1016/j.plantsci.2011.12.013

[pbi12838-bib-0053] Wang, Z. , Fang, H. , Chen, Y. , Chen, K. , Li, G. , Gu, S. and Tan, X. (2014) Overexpression of BnWRKY33 in oilseed rape enhances resistance to Sclerotinia sclerotiorum. Mol Plant Pathol. 15, 677–689. 24521393 10.1111/mpp.12123 PMC6638750

[pbi12838-bib-0054] Willemsen, V. , Wolkenfelt, H. , de Vrieze, G. , Weisbeek, P. and Scheres, B. (1998) The HOBBIT gene is required for formation of the root meristem in the Arabidopsis embryo. Development, 125, 521–531. 9425146 10.1242/dev.125.3.521

[pbi12838-bib-0055] Wu, J. , Zhao, Q. , Yang, Q. , Liu, H. , Li, Q. , Yi, X. , Cheng, Y. *et al* (2016) Comparative transcriptomic analysis uncovers the complex genetic network for resistance to Sclerotinia sclerotiorum in Brassica napus. Sci. Rep. 6:19007. 10.1038/srep19007 PMC4705546 26743436

[pbi12838-bib-0056] Xie, Z. , Zhang, Z.L. , Zou, X. , Huang, J. , Ruas, P. , Thompson, D. and Shen, Q.J. (2005) Annotations and functional analyses of the rice WRKY gene superfamily reveal positive and negative regulators of abscisic acid signaling in aleurone cells. Plant Physiol. 137, 176–189. 15618416 10.1104/pp.104.054312 PMC548849

[pbi12838-bib-0057] Xiong, L. and Yang, Y. (2003) Disease resistance and abiotic stress tolerance in rice are inversely modulated by an abscisic acid‐inducible mitogen‐activated protein kinase. Plant Cell, 15, 745–759. 12615946 10.1105/tpc.008714 PMC150027

[pbi12838-bib-0058] Xu, X. , Chen, C. , Fan, B. and Chen, Z. (2006) Physical and functional interactions between pathogen‐induced Arabidopsis WRKY18, WRKY40, and WRKY60 transcription factors. Plant Cell, 18, 1310–1326. 16603654 10.1105/tpc.105.037523 PMC1456877

[pbi12838-bib-0059] Yan, L. , Liu, Z.‐Q. , Xu, Y.‐H. , Lu, K. , Wang, X.‐F. and Zhang, D.‐P. (2012) Auto‐ and Cross‐repression of Three Arabidopsis WRKY Transcription Factors WRKY18, WRKY40, and WRKY60 Negatively Involved in ABA Signaling. J. Plant Growth Regul. 32, 399–416.

[pbi12838-bib-0060] Yang, B. , Srivastava, S. , Deyholos, M.K. and Kav, N.N.V. (2007) Transcriptional profiling of canola (Brassica napus L.) responses to the fungal pathogen Sclerotinia sclerotiorum. Plant Sci. 173, 156–171.

[pbi12838-bib-0061] Yang, B. , Jiang, Y. , Rahman, M.H. , Deyholos, M.K. and Kav, N.N. (2009) Identification and expression analysis of WRKY transcription factor genes in canola (Brassica napus L.) in response to fungal pathogens and hormone treatments. BMC Plant Biol. 9, 68. 19493335 10.1186/1471-2229-9-68 PMC2698848

[pbi12838-bib-0062] Yoo, S.D. , Cho, Y.H. and Sheen, J. (2007) Arabidopsis mesophyll protoplasts: a versatile cell system for transient gene expression analysis. Nat. Protoc. 2, 1565–1572. 17585298 10.1038/nprot.2007.199

[pbi12838-bib-0063] Yu, D. , Chen, C. and Chen, Z. (2001) Evidence for an important role of WRKY DNA binding proteins in the regulation of NPR1 gene expression. Plant Cell, 13, 1527–1540. 11449049 10.1105/TPC.010115 PMC139550

[pbi12838-bib-0064] Zhao, J. , Wang, J. , An, L. , Doerge, R. , Chen, Z.J. , Grau, C.R. , Meng, J. *et al* (2007) Analysis of gene expression profiles in response to Sclerotinia sclerotiorum in Brassica napus. Planta, 227, 13–24. 17665211 10.1007/s00425-007-0586-z

[pbi12838-bib-0065] Zheng, Z. , Qamar, S.A. , Chen, Z. and Mengiste, T. (2006) Arabidopsis WRKY33 transcription factor is required for resistance to necrotrophic fungal pathogens. Plant J. 48, 592–605. 17059405 10.1111/j.1365-313X.2006.02901.x

[pbi12838-bib-0066] Zhou, N. , Tootle, T.L. and Glazebrook, J. (1999) Arabidopsis PAD3, a gene required for camalexin biosynthesis, encodes a putative cytochrome P450 monooxygenase. Plant Cell, 11, 2419–2428. 10590168 10.1105/tpc.11.12.2419 PMC144139

