# Supplementary material for: Interactions of WRKY15 and WRKY33 transcription factors and their roles in the resistance of oilseed rape to Sclerotinia infection
Source: Plant Biotechnol J. 2017 Nov 9;16(4):911–25. doi: 10.1111/pbi.12838 (PMC5867032; doi:10.1111/pbi.12838)
Supplement: Supplementary file 1 — Figure S1 Map‐based location of BnWRKY33 in the A05 linkage group. Figure S2 Expression levels of partial BnWRKY33‐overexpressing lines (T0 generation). Figure S3 Cis‐elements identified using the BnWRKY33 promoter from rice and the PlantPAN2.0 software. Figure S4 β‐Glucuronidase (GUS) staining of Arabidopsis plants containing P‐346‐GUS or P‐249‐GUS constructs. Figure S5 Amino acid comparison of BnWRKY15 and its homologous protein in Arabidopsis, AtWRKY15. Figure S6 Transcriptional activation of truncated BnWRKY15 and BnWRKY33 genes in Arabidopsis protoplasts. Figure S7 Subcellular localization of the BnWRKY15 and BnWRKY33 genes. Figure S8 β‐Glucuronidase (GUS) histochemical staining and activities of transgenic Arabidopsis plants harbouring P‐15‐GUS. Figure S9 Expression levels of BnWRKY15‐overexpressing lines (T0 generation). Table S1 Lesion areas of both BnWRKY33‐overexpressing and control plants at 48 h (T0) after inoculation with Sclerotinia sclerotiorum. Table S2 Primers used for cloning, plasmid construction and quantitative RT‐PCR. Table S3 Annotation of colonies detected using yeast one‐hybrid assays. [file PBI-16-911-s002.docx]

Table S1 lesion area of *BnWRKY33* overexpression plants and the control infected with *S. Sclerotiorum* for 48hrs (T_0_). *T*-test was calculated by EXCEL and *P* value was list. The wild type Westar was used as control and marked as CK here.

| Sample.NO | lesion areas | | | Mean value | *P* value |
| --- | --- | --- | --- | --- | --- |
| 33-1 | 8.53 | 6.42 | 11.12 | 8.69 | 0.11 |
| 33-2 | 6.08 | 5.41 | 4.86 | 5.45 | 0.02 |
| 33-3 | 12.76 | 4.10 | 7.84 | 8.23 | 0.18 |
| 33-5 | 8.58 | 8.76 | 9.67 | 9.00 | 0.10 |
| 33-6 | 6.20 | 5.06 | 4.28 | 5.18 | 0.01 |
| 33-7 | 7.50 | 4.13 | 7.10 | 6.24 | 0.02 |
| 33-8 | 4.01 | 6.33 | 5.89 | 5.41 | 0.01 |
| 33-10 | 7.09 | 9.79 | 5.14 | 7.34 | 0.05 |
| 33-11 | 5.89 | 7.10 | 6.21 | 6.40 | 0.02 |
| 33-12 | 7.14 | 5.33 | 11.66 | 8.04 | 0.12 |
| 33-13 | 10.75 | 11.65 | 10.63 | 11.01 | 0.41 |
| 33-14 | 6.67 | 6.10 | 11.35 | 8.04 | 0.10 |
| 33-15 | 2.90 | 4.87 | 6.14 | 4.64 | 0.01 |
| 33-16 | 8.18 | 6.55 | 8.83 | 7.85 | 0.04 |
| 33-17 | 7.84 | 7.00 | 7.65 | 7.50 | 0.04 |
| 33-18 | 5.78 | 7.72 | 7.25 | 6.91 | 0.03 |
| 33-20 | 6.45 | 7.07 | 3.85 | 5.79 | 0.01 |
| 33-22 | 3.94 | 10.25 | 9.05 | 7.75 | 0.10 |
| 33-23 | 4.48 | 4.24 | 7.84 | 5.52 | 0.01 |
| 33-24 | 6.08 | 7.67 | 6.38 | 6.71 | 0.02 |
| 33-25 | 4.71 | 10.62 | 3.67 | 6.33 | 0.07 |
| 33-26 | 5.27 | 6.06 | 3.97 | 5.10 | 0.01 |
| 33-27 | 0.00 | 0.73 | 7.64 | 2.79 | 0.03 |
| 33-28 | 4.97 | 6.06 | 5.85 | 5.63 | 0.02 |
| 33-29 | 9.62 | 9.29 | 9.44 | 9.45 | 0.13 |
| 33-32 | 3.78 | 4.63 | 2.59 | 3.67 | 0.01 |
| 33-33 | 7.32 | 6.13 | 6.14 | 6.53 | 0.02 |
| 33-35 | 7.72 | 9.32 | 8.23 | 8.42 | 0.06 |
| 33-36 | 7.02 | 6.18 | 6.33 | 6.51 | 0.03 |
| 33-37 | 6.17 | 5.68 | 4.45 | 5.43 | 0.01 |
| 33-38 | 6.79 | 1.56 | 1.58 | 3.31 | 0.01 |
| 33-39 | 8.75 | 4.44 | 7.56 | 6.92 | 0.03 |
| 33-40 | 3.79 | 4.82 | 6.15 | 4.92 | 0.01 |
| 33-41 | 7.07 | 7.09 | 7.40 | 7.18 | 0.04 |
| 33-42 | 6.58 | 6.58 | 5.27 | 6.14 | 0.02 |
| 33-43 | 4.79 | 4.80 | 1.51 | 3.70 | 0.00 |
| 33-45 | 0.00 | 5.48 | 2.97 | 2.82 | 0.01 |
| 33-44 | 6.79 | 6.15 | 6.40 | 6.45 | 0.03 |
| 33-46 | 6.86 | 5.13 | 3.76 | 5.25 | 0.01 |
| 33-48 | 4.34 | 5.87 | 2.94 | 4.38 | 0.01 |
| 33-49 | 5.68 | 5.27 | 6.81 | 5.92 | 0.02 |
| 33-50 | 6.08 | 6.81 | 6.18 | 6.36 | 0.03 |
| 33-52 | 6.38 | 6.55 | 6.60 | 6.51 | 0.03 |
| 33-54 | 7.33 | 11.91 | 7.31 | 8.85 | 0.14 |
| 33-56 | 7.82 | 8.88 | 9.02 | 8.57 | 0.07 |
| 33-58 | 4.26 | 5.06 | 5.59 | 4.97 | 0.01 |
| 33-59 | 5.68 | 7.10 | 0.79 | 4.52 | 0.02 |
| Control | 13.69 | 10.61 | 9.65 | 11.31 |  |

Table S2 List of primer sequences used for cloning, plasmids construction and qPCR analysis.

| Cloning and Plasmids construction | Sequence |
| --- | --- |
| *BnWRKY33* cloning | TGTATGGCTGCTTCTTCTCTTC |
|  | TCTTCAAGACAAAAACGAATCAA |
| 33-56yh | AGGAGAAAGCGAAaCAAACG |
|  | GACCCGGCACTAGTGTTGTT |
| BnWRKY33 overexpression construct | CGCGGATCCTGTATGGCTGCTTCTTCTCTTC |
|  | CCGAGCTCTCTTCAAGACAAAAACGAATCAA |
| *BnWRKY15* overexpression construct | CGCGGATCCATGGCGGTGGAGCTCATGAC |
|  | CGGGGTACCTCAAGACGATTCCAAAATGAG |
| P-33-GUS | ACGCGTCGACCGTCATACTATTTCTCAAACA |
|  | CGCGGATCCACAGAAAGGGGAATTGTTTTTG |
| P-15-GUS | CCCAAGCTT TGTGATTTCAGCTATAGTATACCCTCTATG |
|  | TCCCCCGGG AAAAGCTCTCTCTACAAGAAGAAGAATG |
| P-346-GUS | ACGCGTCGACCTGACGTAAGCCCGCCTCGTTC |
|  | CGCGGATCCACAGAAAGGGGAATTGTTTTTG |
| P-249-GUS | ACGCGTCGACACTTTTTATCACCTTCTCAG |
|  | CGCGGATCCACAGAAAGGGGAATTGTTTTTG |
| Subcellular location construct for *BnWRKY33* | GACTAGTATGGCTGCTTCTTCTCTTCTTACT |
|  | GACTAGTAGACAAAAACGAATCAAAGAAAGAG |
| Subcellular location construct for *BnWRKY15* | CTAGTCTAGAATGGCGGTGGAGCTCATGACT |
|  | CTAGTCTAGAAGACGATTCCAAAATGAGAT |
| Construct for *BnWRKY15* recombinant proteins expression | CGCGGATCCATGGCGGTGGAGCTCATGAC |
|  | CCGGAATTCTCAAGACGATTCCAAAATGAG |
| Construct for *BnWRKY33* recombinant proteins expression | CGCGGATCCATGGCTGCTTCTTCTCTTCTT |
|  | CGCGAGCTCTCAAGACAAAAACGAATCAA |
| P-346-LUC | ACGCGTCGACCTGACGTAAGCCCGCCTCGTTC |
|  | CGCGGATCCACAGAAAGGGGAATTGTTTTTG |
| P-w2w3-LUC | ACGCGTCGACACACATGGTCTGACCATCTC |
|  | CGCGGATCCACAGAAAGGGGAATTGTTTTTG |
| P-w3-LUC | ACGCGTCGACCTCCTCTCCTCCTCTTCTTCTC |
|  | CGCGGATCCACAGAAAGGGGAATTGTTTTTG |
| P-249-LUC | ACGCGTCGACACTTTTTATCACCTTCTCAG |
|  | CGCGGATCCACAGAAAGGGGAATTGTTTTTG |
| P-Wm1-LUC | ACGCGTCGACCTGACGTAAGCCCGCCTCGTTCAAAGTCTAGGACACATGGTCTGACC |
|  | CGCGGATCCACAGAAAGGGGAATTGTTTTTG |
| P-Wm2-LUC | ACGCGTCGACCTGACGTAAGCCCGCCTCGTTCAAAGTGAAGGACACATGGTCTGACC |
|  | CGCGGATCCACAGAAAGGGGAATTGTTTTTG |
| P-Wm3-LUC | ACGCGTCGACCTGACGTAAGCCCGCCTCGTTCAAAGGCAAGGACACATGGTCTGACC |
|  | CGCGGATCCACAGAAAGGGGAATTGTTTTTG |
| P-Wm4-LUC | ACGCGTCGACCTGACGTAAGCCCGCCTCGTTCAAAATCAAGGACACATGGTCTGACC |
|  | CGCGGATCCACAGAAAGGGGAATTGTTTTTG |
| P-Wm5-LUC | ACGCGTCGACCTGACGTAAGCCCGCCTCGTTCAACCCGGGGGACACATGGTCTGACC |
|  | CGCGGATCCACAGAAAGGGGAATTGTTTTTG |
| P-4W1-LUC | ACGCGTCGACAAAGTCAAGGAAAGTCAAGGAAAGTCAAGGAAAGTCAAGGACACATGGTATCTCCTCTCCTCCTCTTCTTCTCCATCTTCTAATTTTTTCAACACTTTTTATCACCTTCTCAG |
|  | CGCGGATCCACAGAAAGGGGAATTGTTTTTG |
| 15-SK | CGCGGATCCATGGCGGTGGAGCTCATGAC |
|  | CCGGAATTCTCAAGACGATTCCAAAATGAG |
| 33-SK | CGCGGATCCATGGCTGCTTCTTCTCTTCTT |
|  | CGGAATTCTCAAGACAAAAACGAATCAA |
| *BnWRKY15* GAL4BD fusion construct | CGCGGATCCATGGCGGTGGAGCTCATGAC |
|  | CCGGAATTCTCAAGACGATTCCAAAATGAG |
| *BnWRKY33* GAL4BD fusion construct | CGCGGATCCATGGCTGCTTCTTCTCTTCTT |
|  | CGGAATTCTCAAGACAAAAACGAATCAA |
| Fragments inserted into pAbAi | CAGTCAAGGACACATGGTCTGACCATCTCCTCTCCTCCTCTTCTTCTCCATCTTCTAATTTTTTCAACGGTCAAG |
|  | TCGACTTGACCGTTGAAAAAATTAGAAGATGGAGAAGAAGAGGAGGAGAGGAGATGGTCAGACCATGTGTCCTTGACTGAGCT |
| Sequence for probes used in the EMSA experiment |  |
| 33probe | TCAAAGTCAAGGACACATGGTCTGACCATCTCCTCTCCTCCTCTTCTTCTCCATCTTCTAATTTTTTCAACGGTCAAACTT |
| GCC box | CATAAGAGCCGCCACT |
| 33probemut | TCAACCCGGGGGACACATGGTCCCGGGATCTCCTCTCCTCCTCTTCTTCTCCATCTTCTAATTTTTTCAACCCCGGGACTT |
| 33probeW1m1 | TCAAAGTCTAGGACACATGGTCTGACCATCTCCTCTCCTCCTCTTCTTCTCCATCTTCTAATTTTTTCAACGGTCAAACTT |
| 33probeW1m2 | TCAAAGTGAAGGACACATGGTCTGACCATCTCCTCTCCTCCTCTTCTTCTCCATCTTCTAATTTTTTCAACGGTCAAACTT |
| 33probeW1m3 | TCAAAGGCAAGGACACATGGTCTGACCATCTCCTCTCCTCCTCTTCTTCTCCATCTTCTAATTTTTTCAACGGTCAAACTT |
| 33probeW1m4 | TCAAAATCAAGGACACATGGTCTGACCATCTCCTCTCCTCCTCTTCTTCTCCATCTTCTAATTTTTTCAACGGTCAAACTT |
| 33probeW1m5 | TCAACCCGGGGGACACATGGTCTGACCATCTCCTCTCCTCCTCTTCTTCTCCATCTTCTAATTTTTTCAACGGTCAAACTT |
| 3×W1 | TCAAAGTCTAGGACTCAAAGTCTAGGACTCAAAGTCTAGGAC |
| 3×W2 | TCAAAGTGAAGGACTCAAAGTGAAGGACTCAAAGTGAAGGAC |
| 3×W3 | TCAAAGGCAAGGACTCAAAGGCAAGGACTCAAAGGCAAGGAC |
| 3×W4 | TCAAAATCAAGGACTCAAAATCAAGGACTCAAAATCAAGGAC |
| Quantitative PCR |  |
| For *BnWRKY33* | GGGAAGCCATAACCATCC |
|  | TTGTCTGCACAACAATCC |
| For *BnWRKY15* | CTTCACATCATCACCGTCCTTCTG |
|  | CGGCGATATGATTCTCTGAGTTACA |
| For *PAD3* | CCCAGCAGCTCCACTTTTGC |
|  | CGCCATCCCTGGACAGATTC |
| For *CYP71A13* | AGTGGCAGTGGCCAATCTTGTA |
|  | ATCAGAGGAAACTTGCGGCAAA |
| For PR1 | ACGCTCACAACCAAGCACGA |
|  | CCTTGCTTTGCCACATCCAA |
| For PR2 | AGGCCTCGGGATCAAGGTGT |
|  | ACCCTCCGCCTGATTTCTCC |
| For PR3 | ATGCCTTTATCAATGCCGCTAA |
|  | ACTGTCCGTAGTTGTAGTTCCA |
| For PR4 | AAGCCGGTGGTCAGACTTGC |
|  | GCTCCGCCACGTATGGTT |
| For PR5 | GCTCGTACCGGCTGCAACTT |
|  | ACAAGTTTGCGGCGTGGAGT |
| For PDF1.2 | TCACCCTTCTCTTCGCTGCTC |
|  | TGCTCCTTCAAGTCGAATGCAC |
| For reference gene *BnActin2* | CATCCATCGTCCACAG |
|  | ATCATCACAAGCATCCTT |

Table S3 Annotation of colonies detected by yeast-one-hybrid

| Number | *Arabidopsis* Loci | Gene description |
| --- | --- | --- |
| 104-1 | AT1G66580.1 | senescence associated gene 24 (SAG24) |
| 105-2 | AT5G19380.2 | Encodes one of the CRT-Like transporters |
| 111-2 | AT3G06430.1 | Encodes PPR2, a pentatricopeptide repeat protein |
| 116-1 | AT1G61800.1 | Glucose 6-Phosphate/phosphate transporter 2 |
| 119-6 | AT5G04590.1 | A.thaliana gene encoding sulfite reductase |
| 125-1 | AT2G23320.1 | Encodes WRKY DNA-binding protein 15 (WRKY15) |
| 132-1 | AT4G30960.1 | ATCIPK6, CBL-INTERACTING PROTEIN KINASE 6 |
| 136-1 | AT4G30960.1 | ATCIPK6, CBL-INTERACTING PROTEIN KINASE 6 |
| 137-1 | AT5G44070.1 | Phytochelatin synthase gene confers tolerance to cadmium ions |
| 138-1 | AT1G07705.2 | NOT2 / NOT3 / NOT5 family |
| 146-1 | AT2G30860.1 | Encodes glutathione transferase belonging to the phi class of GSTs |
| 148-1 | AT1G56050.1 | GTP-binding protein-related |
| 153-1 | AT3G53260.1 | ATPAL2, encodes phenylalanine lyase |
| 153-2 | AT3G53260.1 | ATPAL2, encodes phenylalanine lyase |
| 164-1 | AT3G53500.2 | Serine/Arginine-Rich Protein Splicing Factors (SR proteins) |
| 169-1 | AT2G45790.1 | Encodes a cytoplasmic phosphomannomutase |
| 172-1 | AT1G14740.1 | Encodes a PHD-finger protein |
| 173-1 | AT5G63570.1 | Encodes a protein with homology to glutamate-1-semialdehyde 2,1-aminomutase |
| 174-3 | AT1G14740.1 | Encodes a PHD-finger protein |
| 182-1 | AT3G52870.1 | IQ calmodulin-binding motif family protein |
| 183-1 | AT4G23290.2 | Encodes a cysteine-rich receptor-like protein kinase |
| 185-1 | AT5G53460.3 | NADH-dependent glutamate synthase The mRNA is cell-to-cell mobile |
| 186-1 | AT3G51160.1 | Catalyzes the first step in the de novo synthesis of GDP-L-fucose |
| 187-1 | AT2G30860.1 | Encodes glutathione transferase belonging to the phi class of GSTs |
| 19-2 | AT3G47470.1 | Encodes a chlorophyll a/b-binding protein that is more similar to the PSI Cab proteins than the PSII cab proteins |
| 192-1 | AT1G14740.1 | Encodes a PHD-finger protein |
| 194-1 | AT1G21100.1 | O-methyltransferase family protein |
| 206-5 | AT3G50670.2 | Encodes U1 snRNP 70K |
| 220-1 | AT5G19350.2 | RNA-binding (RRM/RBD/RNP motifs) family protein |
| 27-1 | AT4G23100.3 | Encodes the enzyme glutamate-cysteine ligase |
| 27-7 | AT5G26280.1 | TRAF-like family protein |
| 28-5 | AT5G54260.1 | DNA repair and meiotic recombination protein, component of MRE11 complex with RAD50 and NBS1 |
| 29-1 | AT4G11830.2 | Encodes one of three phospholipase D enzymes of the gamma class. |
| 3-1 | AT5G26280.1 | TRAF-like family protein |
| 3-15 | AT2G31280.4 | Encodes a LHW-like protein with 80% amino acid identity to LHW |
| 34-1 | AT1G67360.1 | Rubber elongation factor protein (REF) |
| 38-2 | AT1G07705.2 | NOT2 / NOT3 / NOT5 family |
| 42-1 | AT5G13070.1 | MSF1-like family protein; CONTAINS InterPro DOMAIN/s: PRELI/MSF1 (InterPro:IPR006797) |
| 45-1 | AT1G01280.1 | member of CYP703A CYP703A2 is expressed specifically in anthers of land plants |
| 55-3 | AT5G04800.1 | Ribosomal S17 family protein |
| 56-1 | AT3G26200.1 | Putative cytochrome P450 The mRNA is cell-to-cell mobile. |
| 56-6 | [AT3G51160.1](http://www.arabidopsis.org/servlets/TairObject?type=locus&name=AT3G51160) | Catalyzes the first step in the de novo synthesis of GDP-L-fucose. |
| 64-1 | AT3G53500.2 | Serine/Arginine-Rich Protein Splicing Factors (SR proteins) |
| 7-3 | AT3G07780.1 | Encodes a nuclear PHD finger protein |
| 80-1 | AT1G27730.1 | SALT TOLERANCE ZINC FINGER, STZ, ZAT10 |
| 82-2 | AT5G18710.1 | unknown protein |
| 84-1 | AT2G43570.1 | Chitinase, putative (CHI) |
| 85-1 | [AT1G17745.2](http://www.arabidopsis.org/servlets/TairObject?type=locus&name=AT1G17745) | encodes a 3-Phosphoglycerate dehydrogenase |
| 85-2 | AT3G18850.5 | Lysophosphatidyl acyltransferase 5 (LPAT5) |
| 9-2 | AT5G19380.2 | Encodes one of the CRT-Like transporters |
| 96-1 | AT3G18850.5 | Lysophosphatidyl acyltransferase 5 (LPAT5) |
| 99-2 | ATCG01180.1 | chloroplast-encoded 23S ribosomal RNA |


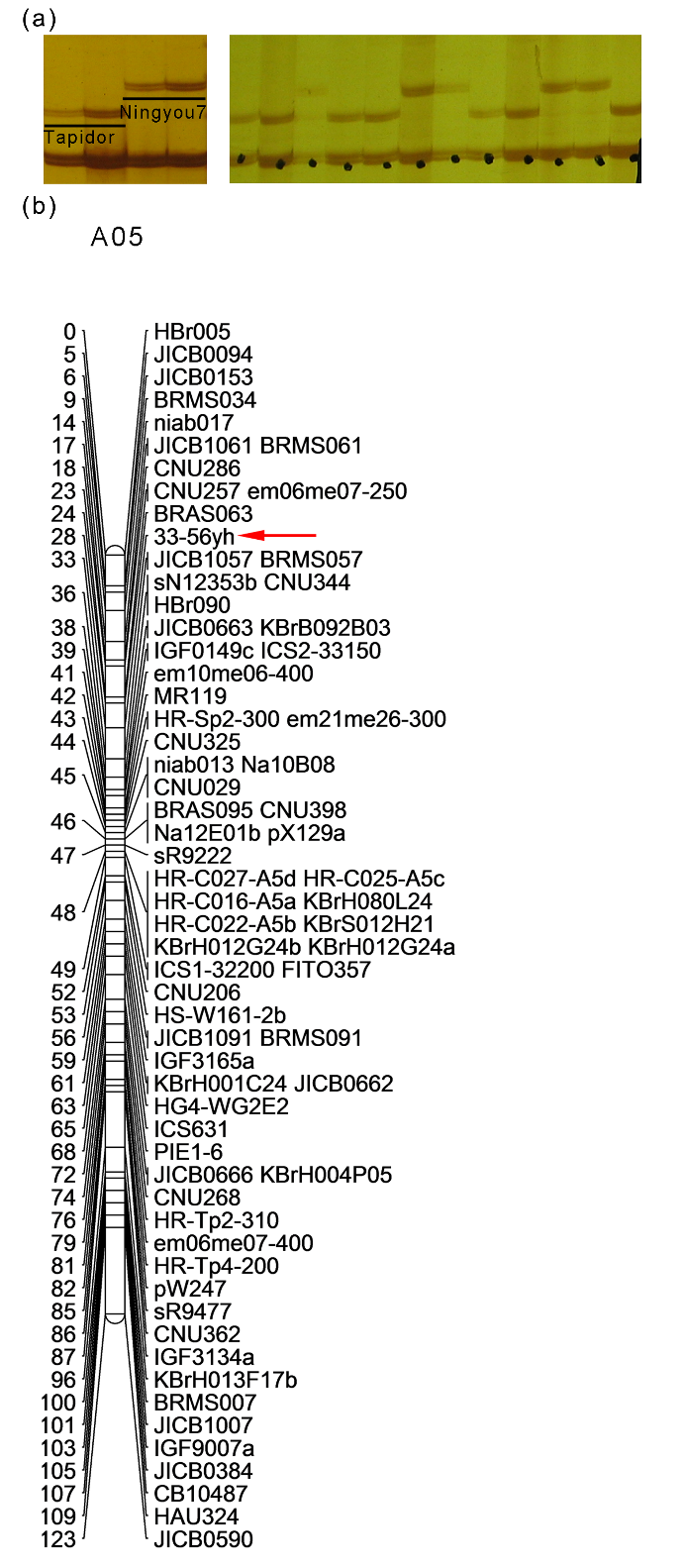


**Figure S1.** Map-based location of *BnWRKY33* in the A05 linkage group. (a) Analysis of the PCR products obtained using 33-56yh on the parental lines, Tapidor and NY7 (left), or individuals (right) of the TN population. (b) *BnWRKY33* was located in the A05 linkage group using the TN population. The red arrow indicates the intron polymorphism marker (33-56yh) developed from the *BnWRKY33* sequence.


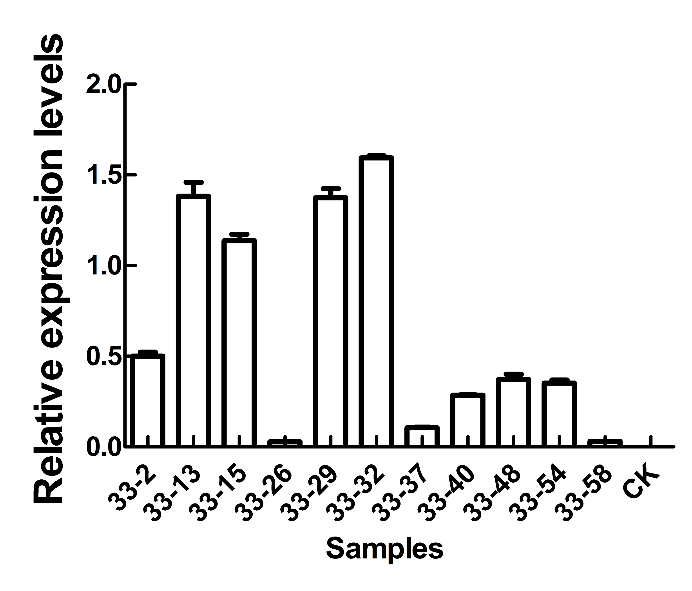


**Figure S2**. Expression levels of partial *BnWRKY33*-overexpressing lines (T_0_ generation). Each fifth leaf of three-week-old plants was sampled for extracting RNA, which was used for subsequent quantitative RT-PCR. CK indicates control (Westar). The data are the means of three technical replicates ± standard errors.


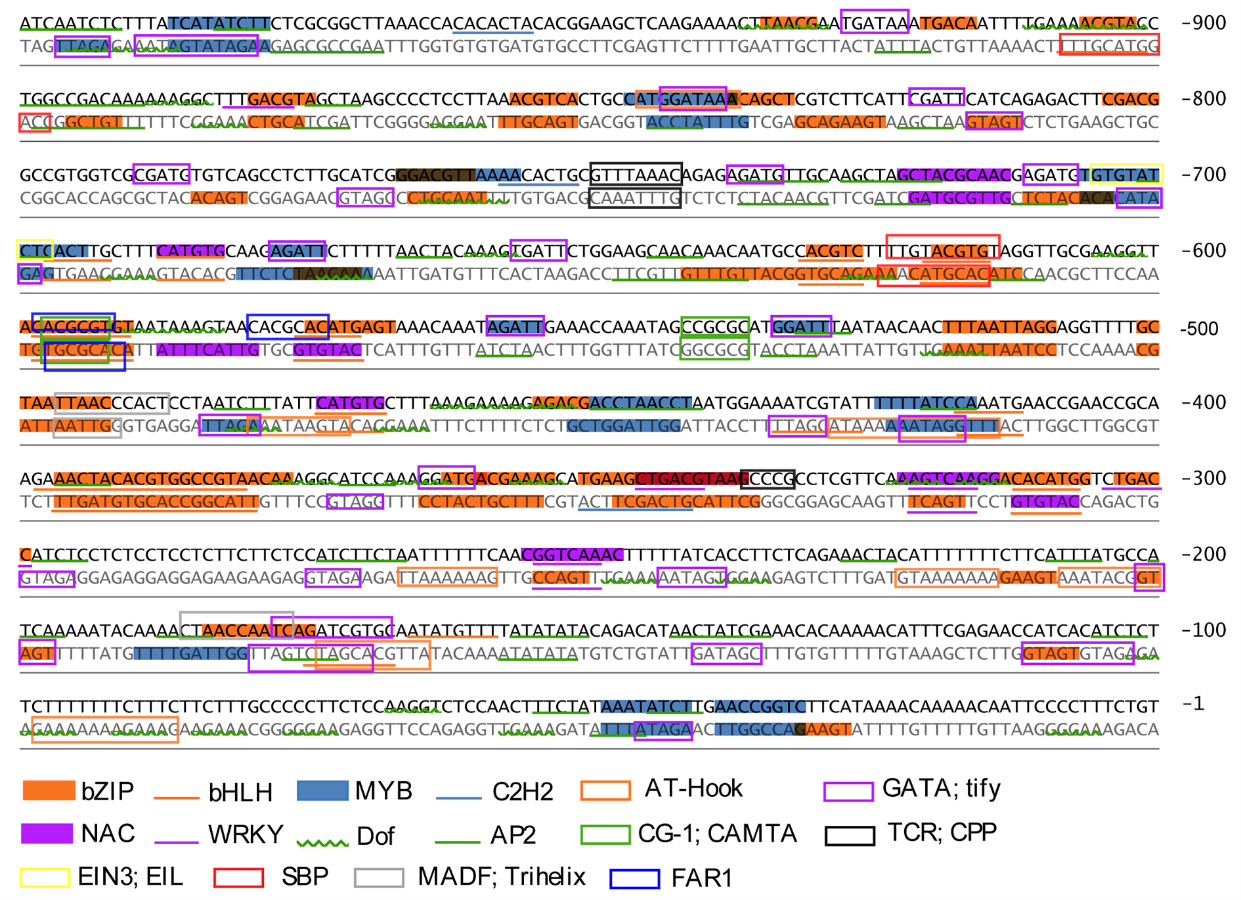


**Figure S3**. *Cis*-elements identified using the *BnWRKY33* promoter from rice and the PlantPAN2.0 software. Different *cis*-elements are indicated by different markers, and each marker along with the name of each *cis*-element predicated in the promoter region is listed under the sequences. The first nucleotide upstream of 5’ ATG was defined as -1. A total length of 1000 bp of the promoter region was used for prediction.


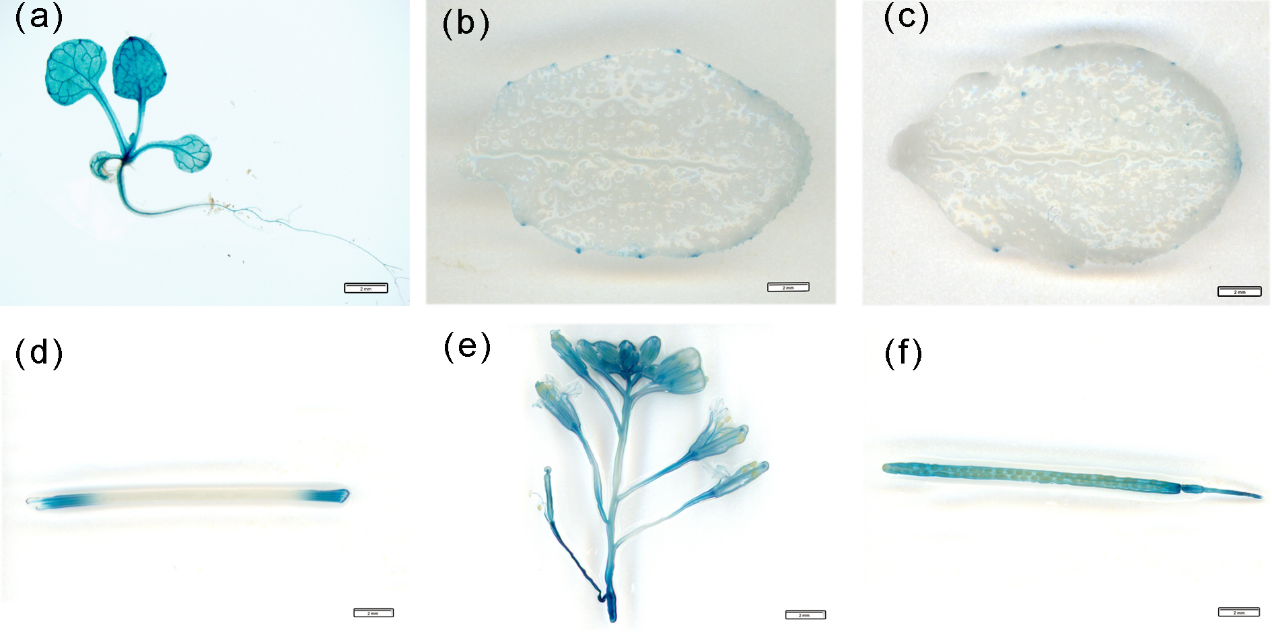


**Figure S4.** β-Glucuronidase (GUS) staining of *Arabidopsis* plants containing P-346-GUS or P-249-GUS constructs. Transgenic *Arabidopsis* plants carrying the P-249-GUS construct were histochemically analyzed using GUS staining of different tissues, including seedlings (a), rosette leaves (b), detached stems (d), flowers and buds (e), and siliques (f). Histochemical analysis of rosette leaves of P-346-GUS transgenic *Arabidopsis* (c). Scale bar, 2 mm.


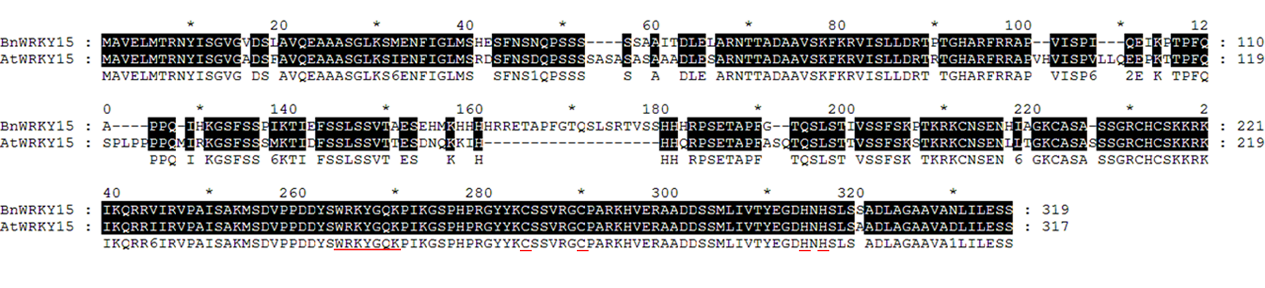


**Figure S5.** Amino acid comparison of BnWRKY15 and its homologous protein in *Arabidopsis*, AtWRKY15. Proteins were aligned using MUSCLE software and displayed using GeneDoc software. Black shading shows identical amino acids. The WRKY zinc-finger domain is marked with red underlining.





**Figure S6.** Transcriptional activation of truncated *BnWRKY15* and *BnWRKY33* genes in *Arabidopsis* protoplasts. (a) Generation of effector constructs using full-length and truncated regions of BnWRKY33. These constructs contained the coding sequences of full-length (BnWRKY33) and truncated regions (33/376, 33/276, 33/176, and 33/76). (b) Generation of effector constructs using full-length and truncated regions of BnWRKY15. These constructs contained the coding sequences of full-length (BnWRKY15) and truncated regions (15/219, 15/119, and 15/19). (c) Reporter plasmids contained five copies of GAL4 binding sites, a minimal TATA region of the cauliflower mosaic virus 35S promoter and the firefly luciferase gene (LUC). (d) Relative luciferase activities in *Arabidopsis* protoplasts co-transformed with effector constructs and reporter genes. The assays were repeated at least three times and showed similar results. The data represent the means ± standard errors of at least three replicates. Significantly different values according to Tukey’s test (ANOVA) are marked with different letters (*P* < 0.05).


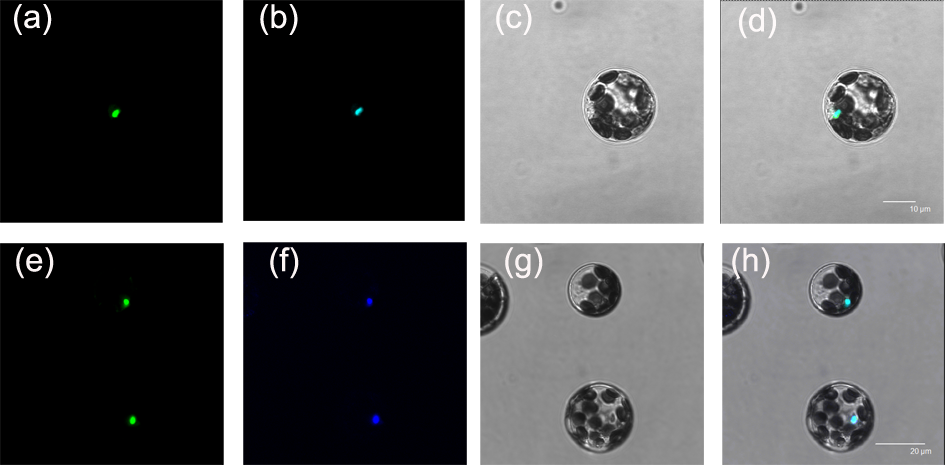


**Figure S7.** Subcellular localization of the *BnWRKY15* and *BnWRKY33* genes. The top and bottom panels indicate the subcellular localization of BnWRKY15 and BnWRKY33, respectively. (a and e) The protoplasts showing a green fluorescent signal at 488 nm. (b and f) The protoplasts showing a cyan fluorescent signal at 561 nm. (c and g) Bright-field images. (d and h) Merged image of the green, cyan and bright fields. Top panel scale bars, 10 µm; bottom panel scale bars, 20 µm.


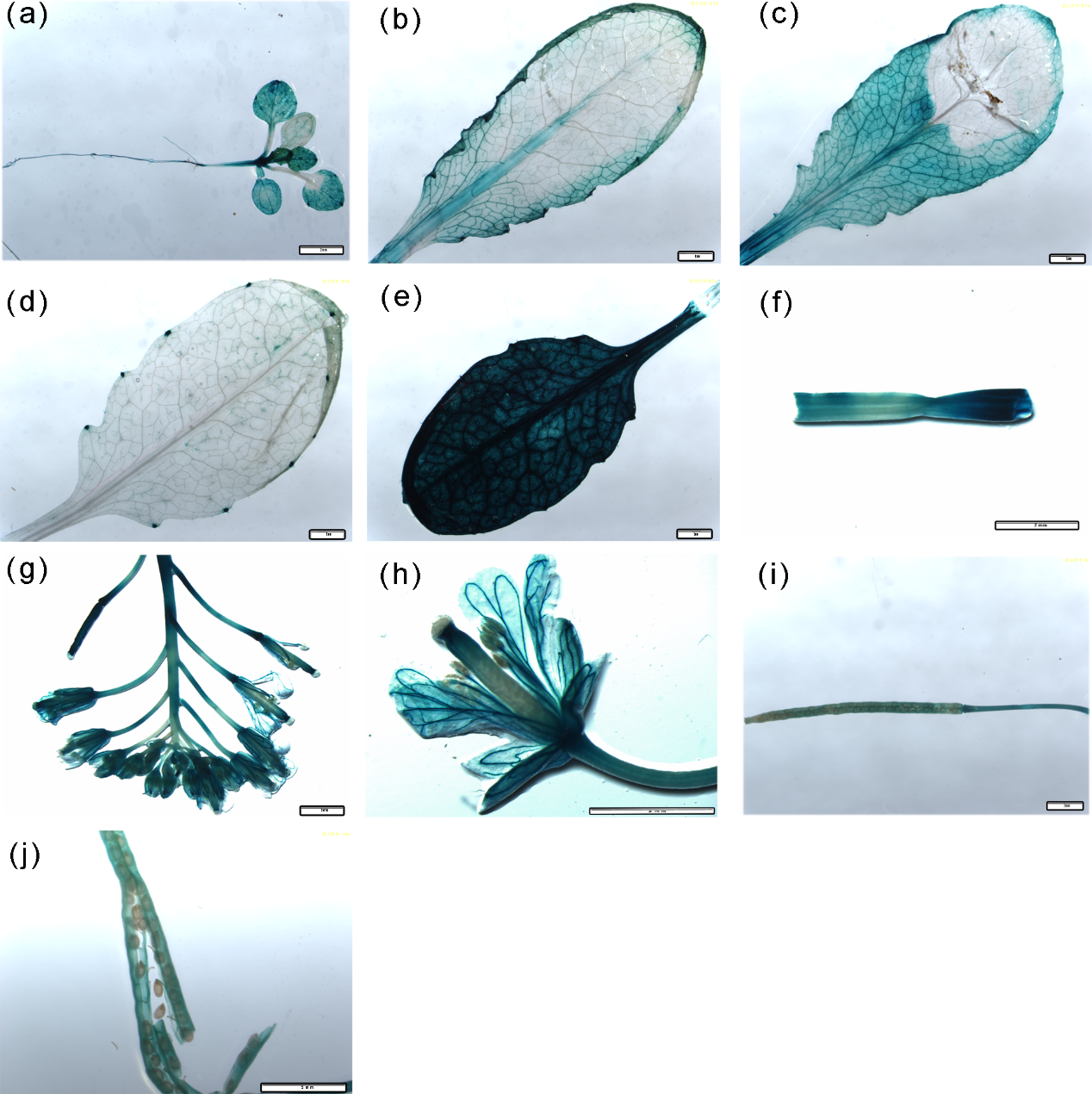


**Figure S8.** β-Glucuronidase (GUS) histochemical staining and activities of transgenic *Arabidopsis* plants harboring P-15-GUS. GUS histochemical staining of two-week-old transgenic seedlings (a), rosette leaves (b), rosette leaves infected with *Sclerotinia sclerotiorum* for 24 h (c), rosette leaves treated with H_2_O_2_ for 0.5 h (d), detached stems (e), flowers and buds (f and g), and (h and i) siliques. Scale bar, 2 mm.


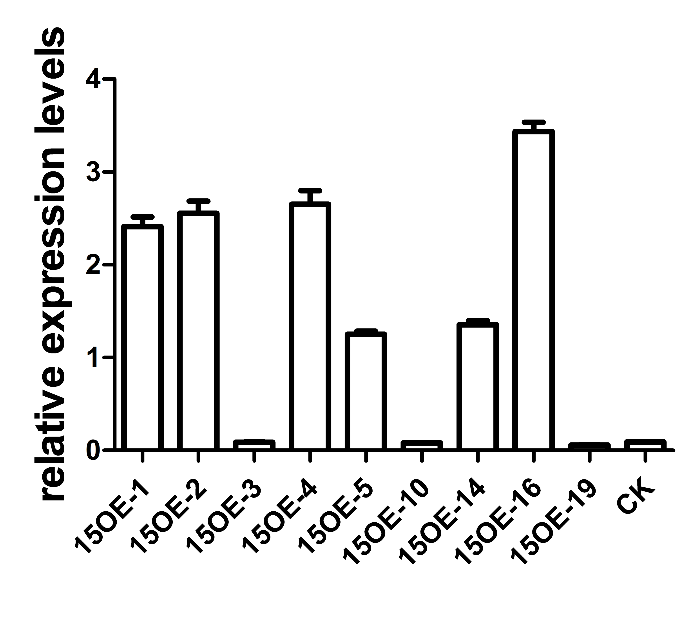


**Figure S9.** Expression levels of *BnWRKY15*-overexpressing lines (T_0_ generation). RNA from each fifth leaf of three-week-old plants was used for quantitative RT-PCR. CK represents control (Westar). The data are the means of three replicates ± standard errors.
