# Supplementary material for: Interactions of WRKY15 and WRKY33 transcription factors and their roles in the resistance of oilseed rape to Sclerotinia infection
Source: Plant Biotechnol J. 2017 Nov 9;16(4):911–25. doi: 10.1111/pbi.12838 (PMC5867032; doi:10.1111/pbi.12838)
Supplement: Supplementary file 2 — Data S1 Methods. [file PBI-16-911-s001.docx]

**Supplementary material: methods**

Plant growth and treatments

Both *A. thaliana* (Col-0) and *B. napus* (Ning RS-1) plants were grown in nutrient-enhanced soil in a growth chamber located at Huazhong Agricultural University, Wuhan city, China (16 h photoperiod, 22 °C day/18 °C night). For the SA and H_2_O_2_ treatments, the leaves of three-week-old plants were sprayed with 1 mM SA (Sigma-Aldrich; pH adjusted to 6.5 by 1 mM KOH) or 100 mM H_2_O_2_*,* respectively. For RNA purification, each fifth leaf of SA-treated plants was harvested at 0, 12, 24, and 48 h after treatment, whereas the leaves of H_2_O_2_-treated plants were harvested at 0, 0.5, 2, and 6 h after treatment. For assaying tissue-specific expression of *BnWRKY*s, different tissues were sampled for RNA extraction. Hypocotyls and cotyledons of one-week-old seedlings were used for seedlings and roots tissue. Each third leaf of two-week-old plants was sampled, but stems and flowers were sampled during peduncle-growing period. Siliques and seeds were collected ten days and thirty days, after flowering, respectively. Furthermore, regarding *S. sclerotiorum* inoculation, potato dextrose agar plugs of *S. sclerotiorum* (provided by Prof. Guoqing Li, State Key Laboratory of Agricultural Microbiology, Huazhong Agricultural University) were placed on the center of the fifth *B. napus* or *A. thaliana* rosette leaf of one-month-old plants, away from leaf veins, and the inoculated plants were placed in a humidity chamber. The leaves were then harvested at 0, 6, 12, 24, and 48 h after inoculation.

Evaluation of resistance to S. sclerotiorum infection

For the resistance assays, each seventh, eighth, and tenth leaf was removed from the plants of three T_0_ lines. Similarly, each fifth leaf of plants from three T_2_ or T_1_ lines that grew for 1 month at 22 °C under a 16-h photoperiod was removed. In addition, both T_0_- and T_1_-generation plants were used for the resistance assays of *BnWRKY15*-overexpressing plants, whereas both T_0_ and T_2_ lines were used for the resistance assays of *BnWRKY33*-overexpressing plants. In each case, the detached leaves with mycelium plugs were placed in a box that contained moistened gauze at the bottom, and the box was covered with transparent film. The box was incubated at 20 ± 2 °C under a 16-h photoperiod. At 48 h after inoculation, the length and width of each lesion were measured and used to calculate the approximate lesion area (π × length × width) (For T_0_ generation); some lesions were measured from images using ImageJ software (For T_1_ and T_2_ generations).

Subcellular protein localization

The full-length ORFs of *BnWRKY15* and *BnWRKY33* were PCR-amplified (Table S2) and inserted into the pM999GFP vector (provided by Dr. Jian Xu, Huazhong Agricultural University) under the control of the CaMV 35S promoter, and a cyan fluorescent protein (CFP) fusion construct containing the Ghd7 protein ([Xue et al., 2008](#_ENREF_59)) under the control of the CaMV 35S promoter was used as a nuclear marker protein. Ten micrograms of each fusion construct was co-transformed into *Arabidopsis* protoplasts using PEG/calcium-mediated transformation, as described by [Yoo et al. (2007)](#_ENREF_63). Transformation details can be found in the supplementary method describing the isolation and transformation of *Arabidopsis* protoplasts. Fluorescent signals were detected and imaged using a confocal laser microscope.

Isolation and transformation of Arabidopsis protoplasts

The isolation and transformation of protoplasts were performed in accordance with the protocol of J. Sheen ([Yoo et al., 2007](#_ENREF_1)), which can be accessed at the website of her laboratory (<http://molbio.mgh.harvard.edu/sheenweb/protocols_reg.html>). *Arabidopsis* plants (Col-0) were grown on autoclaved nutrient-enhanced soil in the growth chamber. The conditions of growth chamber included the following: a short 12 h light (75 µmol/m^2^/s)/12 h dark photoperiod, 23 °C/20 °C light/dark temperature regime and 40–65% relative humidity. Expanded leaves of 4-week-old plants were excised and cut into 0.5–1-mm leaf strips, which were transferred into a prepared enzyme solution that contained 0.4 M mannitol, 20 mM KCl, 20 mM 2-(*N*-morpholino)ethanesulfonic acid (MES) (pH 5.7), 1.5% cellulase R10 (Yakult, Japan) and 0.4% macerozyme R10 (Yakult, Japan). The mixture of leaf strips and enzyme solution was then vacuum infiltrated in the dark for 30 minutes. Afterward, 10 mM CaCl_2_ and 0.1% bovine serum albumin (BSA) was added to the mixture, after which the mixture was digested for another 2.5 h without a vacuum or shaking. Protoplasts in the solution were filtered through a 40-µm nylon mesh into a 50-ml round-bottom tube. An equal volume of W5 solution (154 mM NaCl, 125 mM CaCl_2_, 5 mM KCl, 2 mM MES [pH 5.7]) was added to the solution, after which the solution was centrifuged for 2 minutes at 100 g. Pelleted protoplasts were resuspended gently with 10-20 ml of ice-treated W5 solution. The protoplasts in the W5 solution were placed on ice for 30 minutes and then were pelleted at 100 g. An appropriate volume of MMg solution (0.4 M mannitol, 15 mM MgCl_2_, 4 mM MES [pH 5.7]) was added to resuspend the protoplasts and bring the concentration to 2 × 10^5^/ml. One hundred microliters of protoplasts in the MMg solution was mixed with plasmids and 110 µl of PEG solution (0.4 M mannitol, 15 mM MgCl_2_, 4 mM MES [pH 5.7]), and the mixed transforming solution was incubated at room temperature for 5-30 minutes. Four hundred microliters of W5 solution was added to the transforming solution, after which the solution was centrifuged for 2 minutes at 100 g. Pelleted protoplasts were resuspended using 500 µl of W5 solution and incubated for 12 h. For GFP or CFP plasmids transformation, double volumes of protoplasts, PEG solution and W5 solution were used. Protoplasts were then harvested for microscopic observation of GFP or for assaying the activities of luciferase generated by both the firefly *LUC* gene and the *Renilla* spp. For binding assays of BnWRKYs to promoter regions, three micrograms each of effector plasmids and reporter plasmids was used for co-transformation. For transactivation abilities assay, three micrograms each of effector plasmids and reporter plasmids and 1 µg of internal control plasmids were used for co-transformation. For GFP or CFP plasmids, ten micrograms each was used for transformation. *LUC* gene in accordance with the manufacturer’s instructions (Promega). The *Renilla* *LUC* gene was used as an internal control to normalize firefly *LUC* activities.

References

Yoo, S.D., Cho, Y.H. and Sheen, J. (2007) Arabidopsis mesophyll protoplasts: a versatile cell system for transient gene expression analysis. *Nat Protoc* **2**, 1565-1572.
